# Lectin-Like Bacteriocins from *Pseudomonas* spp. Utilise D-Rhamnose Containing Lipopolysaccharide as a Cellular Receptor

**DOI:** 10.1371/journal.ppat.1003898

**Published:** 2014-02-06

**Authors:** Laura C. McCaughey, Rhys Grinter, Inokentijs Josts, Aleksander W. Roszak, Kai I. Waløen, Richard J. Cogdell, Joel Milner, Tom Evans, Sharon Kelly, Nicholas P. Tucker, Olwyn Byron, Brian Smith, Daniel Walker

**Affiliations:** 1 Institute of Infection, Immunity and Inflammation, College of Medical, Veterinary and Life Sciences, University of Glasgow, Glasgow, United Kingdom; 2 WestCHEM, School of Chemistry, College of Science and Engineering, University of Glasgow, Glasgow, United Kingdom; 3 Institute of Molecular Cell and Systems Biology, College of Medical, Veterinary, and Life Sciences, University of Glasgow, Glasgow, United Kingdom; 4 School of Life Sciences, College of Medical, Veterinary and Life Sciences, University of Glasgow, Glasgow, United Kingdom; 5 Strathclyde Institute for Pharmaceutical and Biomedical Sciences, University of Strathclyde, Glasgow, United Kingdom; Collège de France, France

## Abstract

Lectin-like bacteriocins consist of tandem monocot mannose-binding domains and display a genus-specific killing activity. Here we show that pyocin L1, a novel member of this family from *Pseudomonas aeruginosa*, targets susceptible strains of this species through recognition of the common polysaccharide antigen (CPA) of *P. aeruginosa* lipopolysaccharide that is predominantly a homopolymer of d-rhamnose. Structural and biophysical analyses show that recognition of CPA occurs through the C-terminal carbohydrate-binding domain of pyocin L1 and that this interaction is a prerequisite for bactericidal activity. Further to this, we show that the previously described lectin-like bacteriocin putidacin L1 shows a similar carbohydrate-binding specificity, indicating that oligosaccharides containing d-rhamnose and not d-mannose, as was previously thought, are the physiologically relevant ligands for this group of bacteriocins. The widespread inclusion of d-rhamnose in the lipopolysaccharide of members of the genus *Pseudomonas* explains the unusual genus-specific activity of the lectin-like bacteriocins.

## Author Summary

Due to rapidly increasing rates of antibiotic resistance observed among Gram-negative pathogens, such as *Pseudomonas aeruginosa*, there is an urgent requirement for novel approaches to the treatment of bacterial infections. Lectin-like bacteriocins are highly potent protein antibiotics that display an unusual ability to kill a select group of bacteria within a specific genus. In this work, we show how the lectin-like protein antibiotic, pyocin L1, can kill *Pseudomonas aeruginosa* with extraordinary potency through specific binding to the common polysaccharide antigen (CPA) of *P. aeruginosa* lipopolysaccharide. The CPA is predominantly a homopolymer of the sugar d-rhamnose that although generally rare in nature is found frequently as a component of the lipopolysaccharide of members of the genus *Pseudomonas*. The targeting of d-rhamnose containing polysaccharides by pyocin L1 and a related lectin-like protein antibiotic, putidacin L1, explains the unusual genus- specific killing activity of the lectin-like bacteriocins. As we learn more about the link between changes to the microbiome and a range of chronic diseases there is a growing realisation that the ability to target specific bacterial pathogens while maintaining the normal gut flora is a desirable property for next generation antibiotics.

## Introduction

The ability to target a subgroup of pathogenic bacteria in a complex bacterial community has potential applications in medicine and agriculture where the maintenance of a ‘normal’ microbiome is beneficial. For example, the use of broad spectrum antibiotics to treat bacterial infections is known to cause a range of complications associated with collateral damage to the microbiome, including antibiotic associated diarrhea and *Clostridium difficile* infection [1], [2]. In addition, there is growing evidence to suggest that microbial dysbiosis may play a role in a range of chronic diseases such as inflammatory bowel disease, diabetes, obesity and rheumatoid arthritis [3], [4], [5], [6]. Indeed, for Crohn's disease, where the link with dysbiosis is well established, the administration of multiple courses of antibiotics is associated with an increased risk factor for the development of this chronic form of inflammatory bowel disease [7], [8], [9].

In contrast to the broad spectrum antibiotics that are widely used in medicine and agriculture, protein antibiotics known as bacteriocins often target a specific bacterial species or a group of closely related bacterial species [10], [11], [12], [13]. Well characterised bacteriocins include the S-type pyocins from *P. aeruginosa* and the closely related colicins of *E. coli* [12], [13]. The colicin-like bacteriocins form a diverse family of multidomain protein antibiotics which share similar mechanisms of uptake and kill cells through either a pore-forming activity, a specific nuclease activity against DNA, tRNA or rRNA or through inhibition of cell wall synthesis [14], [15], [16], [17]. In the case of S-type pyocins it is thought that their activity is limited to strains of *P. aeruginosa*, whereas colicins show activity against *E. coli* and some strains of closely related bacteria such as *Salmonella* spp. [18]. In the case of colicins and S-type pyocins, killing specificity is primarily determined by the presence of a specific outer membrane receptor on the cell surface. For example, the well characterised E group colicins utilise the TonB-dependent BtuB receptor, which has a normal physiological role in vitamin B_12_ uptake [19]. Colicin-like bacteriocins have also been shown to have a potent antibiofilm activity, indicating their potential as useful therapeutics for the treatment of chronic biofilm mediated infections [20], [21]. In the case of the opportunistic human pathogen *P. aeruginosa* there is an urgent requirement for the development of novel therapeutic options since its ability to form drug-resistant biofilms in combination with the presence of an outer membrane that is highly impermeable to many classes of antibiotics can make this pathogen essentially untreatable in some groups of patients. This is exemplified in cystic fibrosis patients where chronic lung infection with *P. aeruginosa* is the leading cause of mortality [22].

An interesting addition to this group of protein antibiotics is the recently discovered lectin-like bacteriocins that contain two carbohydrate-binding domains of the monocot mannose-binding lectin (MMBL) family [23], [24], [25], [26], [27]. Lectin-like bacteriocins from *P. putida* (putidacin L1 or LlpA_BW_) *P. syringae* (LlpA_Pss642_) and *P. fluorescens* (LlpA1_Pf-5_) have been characterised and have the unprecedented ability to kill strains of a broad range of bacterial species within the genus *Pseudomonas*, but are not active outside this genus [24], [26], [27]. Similarly the lectin-like bacteriocin LlpA_Xcm761_ from *Xanthomonas citri* pv. *malvacearum* LMG 761 has the ability to kill various species within the genus *Xanthomonas* [24]. The molecular basis of this unusual genus specific activity has not been explained.

Lectins are a structurally and evolutionarily diverse class of proteins produced widely by prokaryotes and eukaryotes and are defined by their ability to recognise and bind carbohydrates. This binding is generally highly specific and mediates a range of diverse functions, including cell-cell interaction, immune recognition and cytotoxicity [28], [29] MMBLs represent a structurally conserved lectin subclass, of which the mannose-binding *Galanthus nivalis* agglutinin (GNA) was the first to be characterised [30]. The MMBL-fold consists of a three sided β-prism; each face of which contains a sugar binding motif with the conserved sequence QxDxNxVxY [31]. While originally identified in monocots like *G. nivalis* or *Allium sativum*, it is now recognised that proteins of this class are distributed widely throughout prokaryotes and eukaryotes, where they have evolved to mediate diverse functions [30], [32], [33], [34]. Structural and biochemical analysis of MMBLs has shown that they are generally translated as a single polypeptide chain containing tandem β-prism domains that are then proteolytically processed into monomers. These domains often form homo- or hetero-dimers by strand exchange and π-stacking [35].

The lectin-like bacteriocins are not proteolytically processed and thus consist of a single peptide chain, containing tandem β-prism domains. Sequence alignments of members of this class from *Pseudomonas* spp. show complete conservation of two sugar binding motifs on the C-terminal domain and partial conservation of two sites on the N-terminal domain [23]. Recent work by Ghequire *et al* [23] on the characterisation of putidacin L1 shows these motifs to be important for cytotoxicity. Mutagenesis of the first C-terminal motif has the most dramatic effect on activity, while mutagenesis of the second C-terminal and first N-terminal sugar binding motifs leads to a synergistic reduction in activity. This study also showed low-affinity binding between putidacin L1 and methyl-α-d-mannose or a range of mannose containing oligosaccharides. However, K_d_s for these protein-carbohydrate complexes were reported in the range from 46 mM for methyl-α-d-mannoside to 2 mM for a mannose containing pentasaccharide [23]. An extensive search for high affinity carbohydrate binding through the use of glycan arrays failed to detect high affinity carbohydrate binding for this lectin-like bacteriocin [23].

Despite progress in our understanding of the structure and host range of MMBL-like bacteriocins, the mechanism by which these bacteriocins target susceptible strains and exert their antimicrobial effects is unknown. Here we report on the discovery of a novel member of this family, pyocin L1 from *P. aeruginosa*, and show that it utilises lipopolysaccharide (LPS) as a surface receptor, specifically targeting the common polysaccharide antigen (CPA) that is a conserved homopolymer of d-rhamnose. Structural and biophysical analysis shows that the C-terminal carbohydrate binding motifs are responsible for d-rhamnose recognition and that these sites are specific for this sugar over d-mannose. Further to this, we show that the previously described putidacin L1 also selectively binds LPS from susceptible, but not from resistant, *P. syringae* isolates and shows selectivity for d-rhamnose over d-mannose. This work shows that the physiologically relevant ligand for the QxDxNxVxY carbohydrate binding site of the lectin-like bacteriocins is indeed d-rhamnose and not d-mannose as previously thought. As such, the genus-specific activity of lectin-like bacteriocins from *Pseudomonas spp.* can be attributed to the widespread inclusion of the rare d-rhamnose in the LPS of members of the genus *Pseudomonas*.

## Results

### Identification and characterisation of pyocin L1

As part of a wider project, aimed at identifying bacteriocins that could be used as novel therapeutics in the treatment of *P. aeruginosa* infections, we searched the genomes of 10 recently sequenced clinical and environmental isolates of *P. aeruginosa* for genes with homology to known bacteriocins. One putative bacteriocin gene identified in strain C1433, an isolate from a patient with cystic fibrosis, encodes a protein with 31% identity to the lectin-like bacteriocin LlpA1_Pf-5_, from *P. fluorescens*. This protein, designated pyocin L1, contained 256-amino acids with a predicted molecular mass of 28413 Da. Alignment of the pyocin L1 protein sequence with other lectin-like bacteriocins, LlpA1_Pf-5_, LlpA_Pss642_, putidacin L1 (LlpA_BW_), LlpA_Au1504_ from *Burkholderia cenocepacia* and LlpA_Xcm761_ from *Xanthomonas citri* pv. *malvacearum* shows that pyocin L1 contains tandem MMBL domains with three conserved QxDxNxVxY MMBL sugar-binding motifs (Figure S1). Two of these motifs are located in the C-terminal domain of the protein and one in the N-terminal domain. Comparison with the sequences of other lectin-like bacteriocins shows that the C-terminal QxDxNxVxY motifs are highly conserved, with only LlpA_Xcm761_ lacking one C-terminal motif. In contrast the N-terminal sugar-binding motifs are less well conserved with only LlpA_Au1504_ possessing two fully conserved QxDxNxVxY motifs (Figure S1).

In order to determine the killing spectrum of pyocin L1 we cloned the pyocin L1 open reading frame into the pET21a vector and expressed and purified the protein by nickel affinity, anion exchange and size exclusion chromatography. Purified pyocin L1 was tested for its ability to inhibit the growth of 32 environmental and clinical isolates of *P. aeruginosa* using an overlay spot plate method on LB agar [36]. Under these conditions, pyocin L1 showed killing activity against nine of the *P. aeruginosa* strains tested. Strain E2, an environmental isolate from a tomato plant for which the genome sequence is available, and strain P8, a clinical isolate from a cystic fibrosis patient, showed the greatest sensitivity to pyocin L1 with killing observed down to concentrations of 27 nM and 7 nM, respectively. Pyocin L1 also showed activity against 5 of the 11 *P. syringae* strains tested, although the effect was much weaker, with cell killing observed at high µM concentrations.

### Pyocin L1 targets the common polysaccharide antigen (CPA) of *P. aeruginosa* LPS

**Figure 1:**
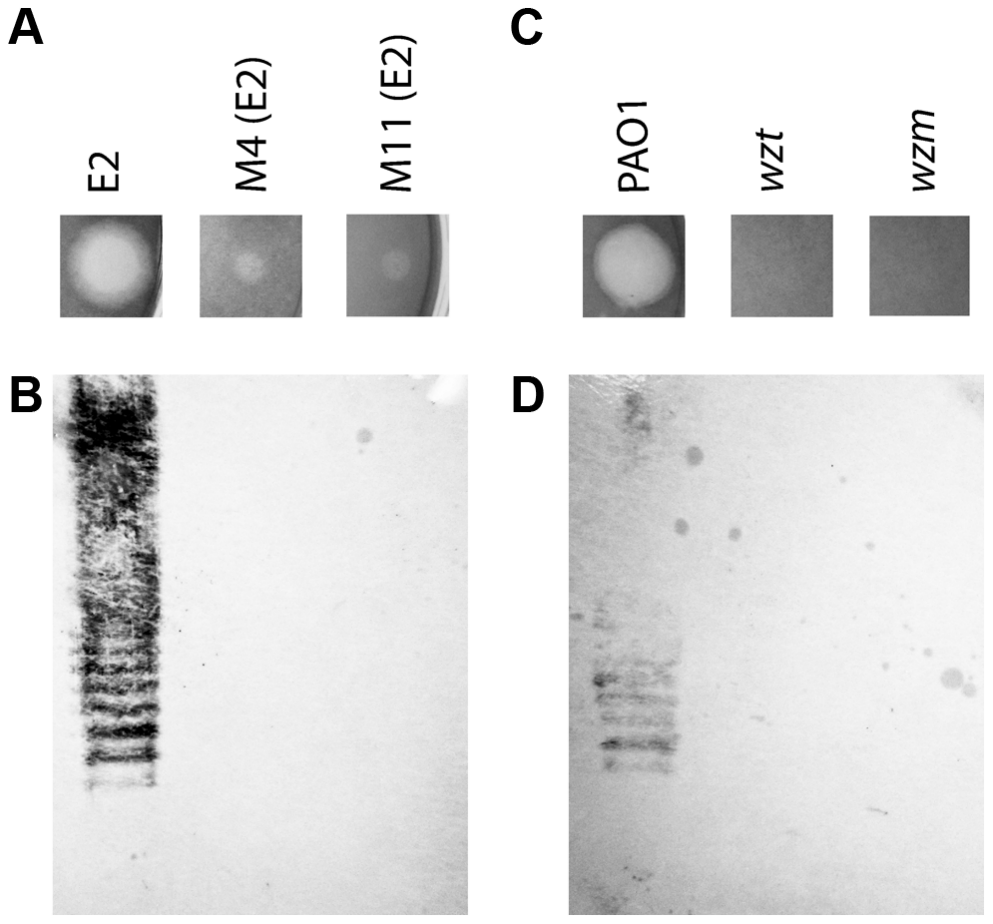
CPA production correlates with pyocin L1 killing. (A) Inhibition of growth of *P. aeruginosa* E2 and tolerant mutants M4 and M11 by pyocin L1, as shown by a soft agar overlay spot-test. 5 µl of purified pyocin L1 (1.5 mg ml^−1^) was spotted onto a growing lawn of cells. Clear zones indicate cell death. (B) Expression of CPA by *P. aeruginosa* E2 and tolerant mutants, visualised by immunoblotting with the CPA specific antibody N1F10. (C) Inhibition of growth of *P. aeruginosa* PAO1 and PAO1 *wzm* and *wzt* mutants by pyocin L1 (details as for A). (D) Expression of CPA by PAO1 and *wzm* and *wzt* strains (details as for B).

In order to gain insight into the bacteriocidal activity of pyocin L1, we subjected *P. aeruginosa* E2 to high concentrations of recombinant protein and recovered mutants with greatly increased tolerance to pyocin L1 (Figure 1A). The genomes of two of these mutants were sequenced and comparative analysis with the genome of wild-type E2 revealed a dinuclear deletion, C710 and T711, of the 1146-bp *wbpZ* gene. This deletion was common to both mutants. *wbpZ* encodes a glycosyltransferase of 381 amino acids that plays a key role in lipopolysaccharide synthesis, specifically in the synthesis of the common polysaccharide antigen (CPA) also known as A-band LPS [37], (Figure S2). Most strains of *P. aeruginosa* produce two distinct LPS-types that differ in their O-antigen, but share the same core oligosaccharide. The CPA is predominantly a homopolymer of d-rhamnose and the O-specific antigen contains a heteropolymeric repeating unit that varies widely among strains [38]. Consistent with mutation of *wbpZ*, we found that production of CPA, as determined by immunoblotting with a CPA-specific monoclonal antibody [39], in both M4(E2) and M11(E2) was reduced to undetectable levels (Figure 1B). Visualisation of LPS from these strains was performed via silver staining and comparable quantities of LPS were shown to be present. These observations suggest that pyocin L1 may utilise CPA as a cellular receptor. To test this idea further, we obtained two transposon insertion mutants of *P. aeruginosa* PAO1, which is sensitive to pyocin L1, with insertions in the genes responsible for the transport of CPA to the periplasm [40]. These two genes, *wzt* and *wzm*, encode the ATP-binding component and membrane component of a CPA dedicated ABC transporter [38]. Pyocin L1, which shows good activity against PAO1 showed no activity against strains with insertions in *wzm* and *wzt* (Figure 1C) and immunoblotting with a CPA-specific antibody confirmed the absence of the CPA in these pyocin L1 resistant strains (Figure 1D). Thus, the presence of CPA on the cell surface is required for pyocin L1 killing.

**Figure 2:**
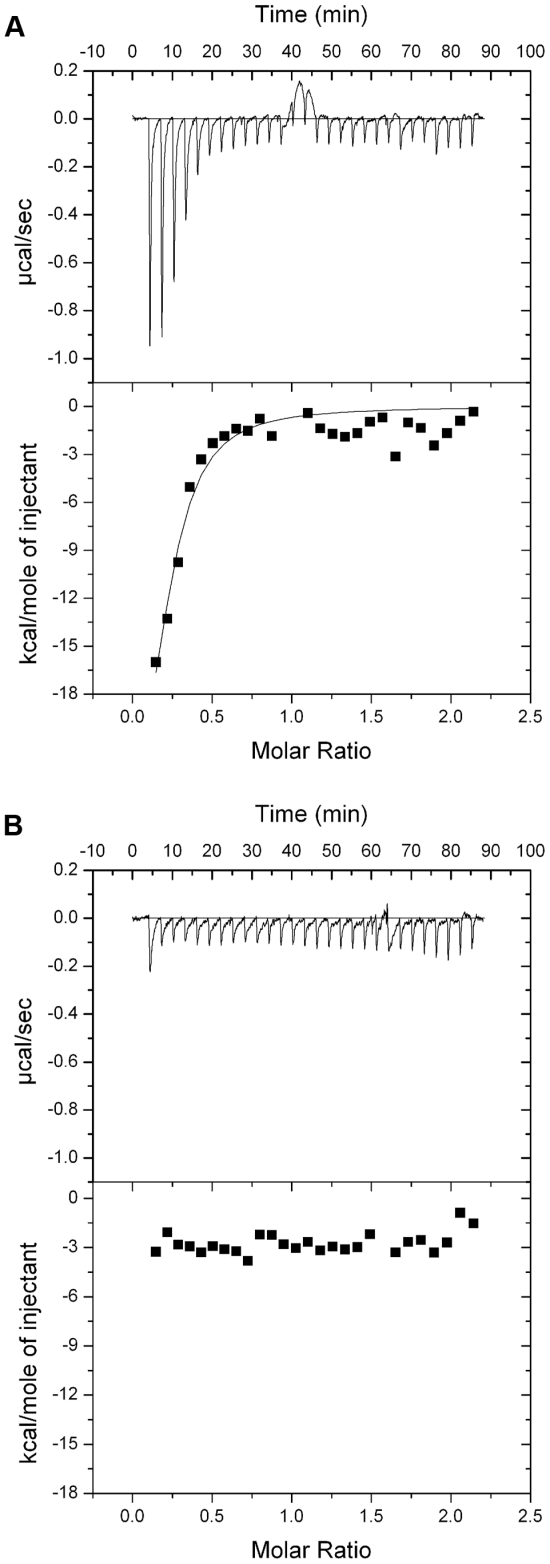
Pyocin L1 binds strongly to CPA from *P. aeruginosa* PAO1. (A) ITC binding isotherm of pyocin L1 (150 µM) titrated into isolated LPS-derived polysaccharide (1 mg ml^−1^) from wild-type *P. aeruginosa* PAO1. Strong, saturable heats were observed indicative of a strong interaction. Curve fitted with a single binding site model. (B) ITC isotherm of pyocin L1 (150 µM) titrated into isolated LPS-derived polysaccharide (1 mg ml^−1^) from PAO1 *wzt*. No saturable binding isotherm was observed.

In order to determine if the requirement for CPA is due to a direct interaction with pyocin L1 we purified LPS from wild-type PAO1 and from the pyocin L1 resistant, *wzm* and *wzt* mutants (which produce no CPA but do produce the O-specific antigen) and analysed the pyocin-CPA interaction by isothermal titration calorimetry (ITC). Titration of pyocin L1 into isolated LPS-derived polysaccharides (a mixture of CPA and the O-specific antigen containing polysaccharides) from PAO1 gave rise to strong saturable exothermic heats of binding (Figure 2A), whereas no binding was detected on titration of pyocin L1 into an equivalent concentration of LPS carbohydrates from PAO1 *wzt*, which produces the O-specific antigen but not the CPA (Figure 2B). These data show that pyocin L1 binds directly to the CPA and that this interaction is required for killing. The CPA is therefore likely to be the cellular receptor for pyocin L1.

### Pyocin L1 binds the monosaccharide d-rhamnose

The evolutionary relationships between MMBL-like bacteriocins and the originally identified mannose-binding members of this protein family, led to the assumption that carbohydrate binding of polysaccharides by the lectin-like bacteriocins is primarily mediated through binding of d-mannose at one or more of their conserved QxDxNxVxY carbohydrate binding motifs. Indeed, the recent structures [23] of putidacin L1 bound to mannose-containing monosaccharides adds weight to this idea, although measured affinities between polysaccharides and putidacin L1 are weak (mM) and so may not be physiologically relevant. However, the strong interaction between pyocin L1 and CPA, is incompatible with this and suggests that d-rhamnose and not d-mannose is the likely physiological substrate for the QxDxNxVxY carbohydrate binding motifs.

**Figure 3:**
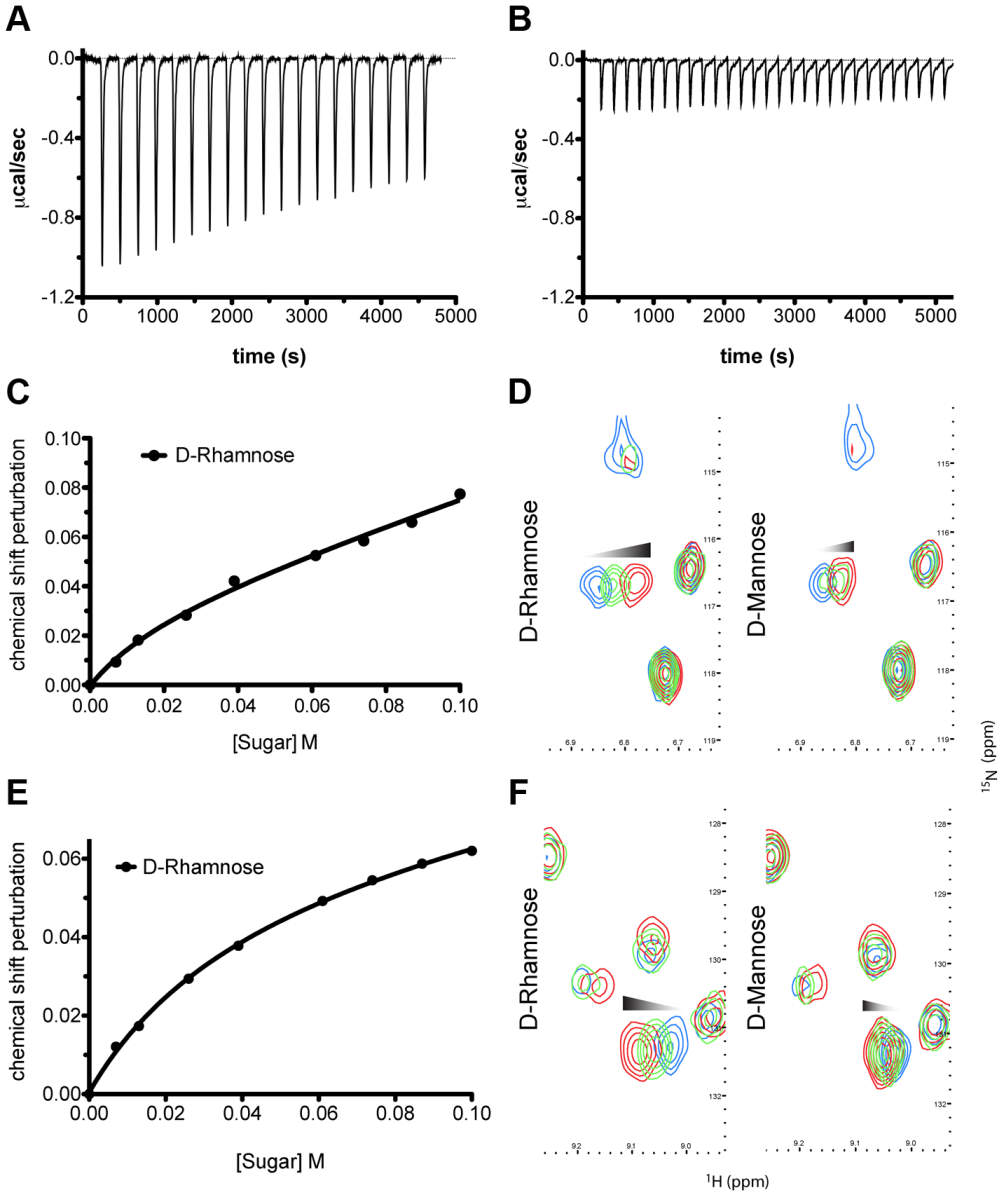
Pyocin L1 shows specificity for d-rhamnose compared with d-mannose. (A) ITC binding isotherm of d-rhamnose (50 mM) titrated into pyocin L1 (100 µM). Weakly saturable heats were observed, indicative of binding with modest affinity (Kd ∼5–10 mM). (B) ITC binding isotherm of d-mannose (50 mM) titrated into pyocin L1 (100 µM). Small-weakly saturable heats were observed, indicative of very weak interaction (Kd ∼50 mM). Titration of monomeric sugars into ^15^N-labelled pyocin L1, monitored using ^1^H-^15^N HSQC NMR spectroscopy. Shifts within spectra were converted to chemical shift perturbation (CSP) values using equation Δ_ppm_ = √ [Δδ_HN_+(Δδ_N_*α_N_)^2^]. CSP values are plotted against sugar concentration in (C) and (E) and visualised in (D) and (F). Peak positions, which correspond to backbone amide signals, at selected sugar concentrations (blue: no sugar, green: 60 mM, red: 100 mM) are shown. Perturbation of peak position (ppm) is indicative of association between ligand and protein molecules in solution.

To determine the affinity of pyocin L1 for d-rhamnose and d-mannose, isothermal titration calorimetry (ITC) was performed. Titration of pyocin L1 into d-rhamnose gave rise to weakly saturable heats of binding that are significantly larger than the heats observed on titration of pyocin L1 into an identical concentration of d-mannose (Figure 3). From this experiment an apparent K_d_ of 5–10 mM was estimated for the interaction of pyocin L1 with d-rhamnose with apparently weaker binding for d-mannose, K_d_>50 mM. The interaction between pyocin L1 and these monosaccharides was also probed using NMR with ^15^N labelled pyocin L1, monitoring changes to its ^15^N-heteronuclear single quantum correlation (^15^N-HSQC) spectra on addition of d-rhamnose or d-mannose. In the absence of added monosaccharide ^15^N-HSQC spectra of pyocin L1, which should contain one crosspeak for each non-proline amide NH as well as peaks for the NH groups in various side chains, were well resolved and dispersed, indicative of a folded protein. Chemical shift perturbation monitored by ^15^N-HSQC allows the mapping of changes to a protein that occur on ligand binding. Addition of either d-rhamnose or d-mannose up to a concentration of 100 mM did not give rise to large or global changes in chemical shifts (Figure S3). On addition of d-rhamnose significant chemical shift changes were observed for a discrete subset of peaks including some in the amide side chain region of the spectra, while changes of a smaller magnitude were observed on the addition of equal concentrations of d-mannose (Figure S3). Fitting the chemical shift changes that occur on addition of d-rhamnose, for peaks showing strong shifts, to a single site binding model indicates a K_d_ for the pyocin L1- d-rhamnose complex in the range of 5–20 mM (Figures 3C–F). These data correlated well with the ITC sugar binding data, with low mM binding of pyocin L1 to d-rhamnose and much weaker binding to d-mannose.

### d-rhamnose and the CPA bind to the C-terminal QxDxNxVxY motifs of pyocin L1

**Figure 4:**
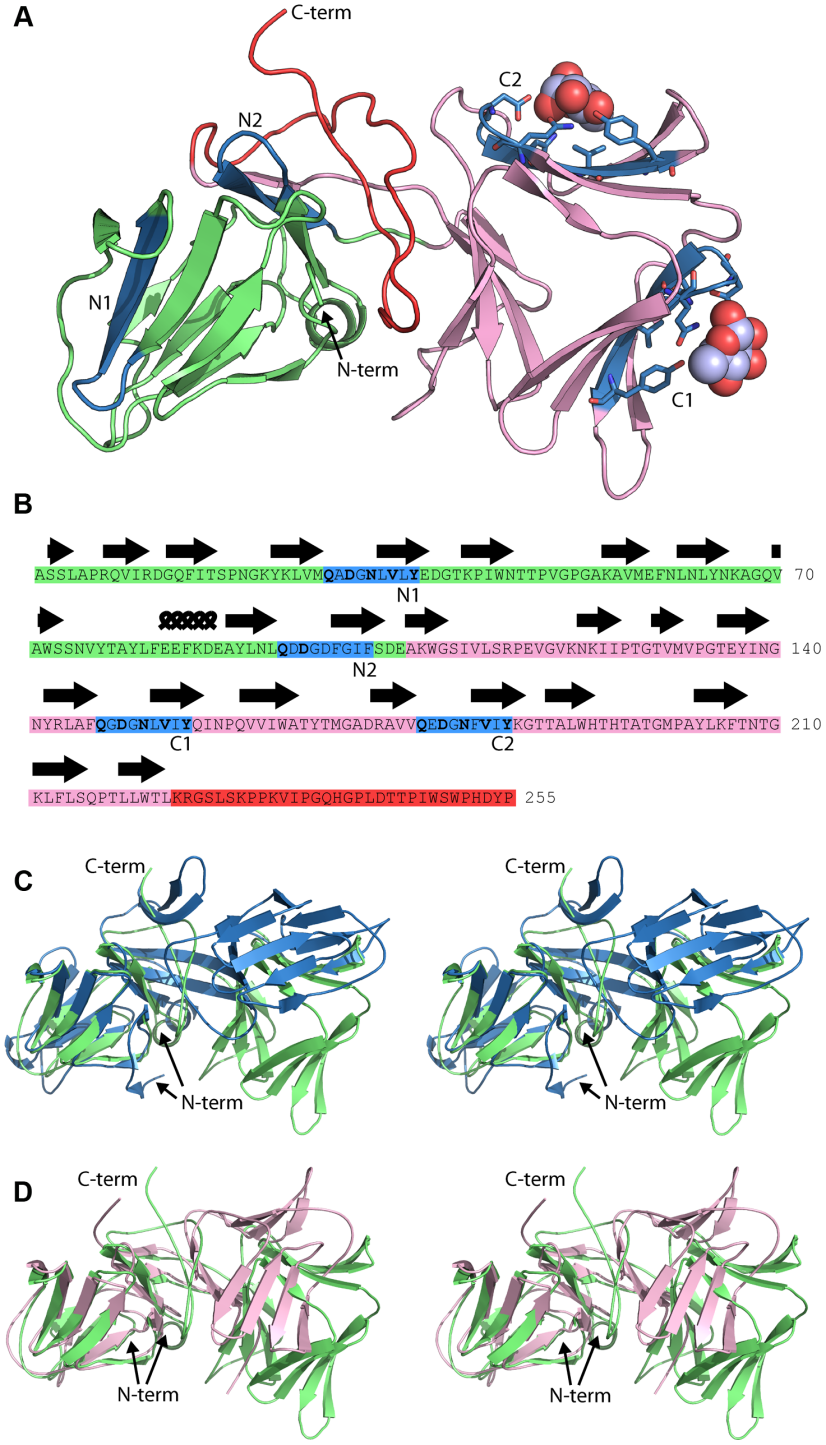
Crystal structure of pyocin L1 reveals tandem MMBL domains and sugar-binding motifs. (A) Ribbon diagram of structure of pyocin L1 in complex with α-d-rhamnose, amino acids 2-256. N-terminal domain (green), C-terminal domain (pink), C-terminal extension (red), α-d-rhamnose (spheres) and sugar binding sites containing the conserved or partially conserved QxDxNxVxY motif are highlighted (blue) and are designated N1, N2 and C1, C2 according to order of appearance in the primary sequence of the N- and C-terminal domains, respectively. Pyocin L1 residues involved in hydrogen bonding with α-d-rhamnose are shown in stick representation. (B) Sequence and secondary structure (β-sheets = arrows, α-helices = coils) of pyocin L1 with colours corresponding to the structure in (A). Residues conserved in sugar binding motifs are shown in bold. (C) Structural alignment of pyocin L1 (green) and putidacin L1 (blue) based on N-terminal MMBL domain in wall-eyed stereo. (D) Structural alignment of pyocin L1 (green) and *Allium sativum* agglutinin (1BWU) (pink) based on N-terminal MMBL domain in wall-eyed stereo.

In an attempt to determine the location of the pyocin L1 d-rhamnose binding site(s) and the structural basis of the d-rhamnose specificity of pyocin L1 we determined the X-ray structures of pyocin L1 with bound d-mannose, d-rhamnose and in the unbound form (Table 1). Pyocin L1, as predicted by sequence homology to MMBL proteins, consists of two tandem β-prism domains characteristic of MMBLs, connected by antiparallel strands propagating from the end of each MMBL domain and lending a strand to the reciprocal β-prism. The strands contain a tryptophan residue which forms π-stacking interactions with two other tryptophans in the β-prism to stabilise the structure (Figure 4A). This interaction is conserved throughout MMBLs, with most members of the class utilising it to form either homo- or hetero-dimers of single MMBL subunits. However, in pyocin L1, as with the recently described structure of putidacin L1, both domains are from a single polypeptide chain [23]. Other structural elements are also common between the two bacteriocins, namely a C-terminal extension of 30 amino acids and a two-turn α-helix insertion into loop 6 of the N-terminal MMBL domain (Figure 4B). The overall root mean square deviation (rmsd) of backbone atoms for pyocin L1 and putidacin L1 is 7.5 Å, which is relatively high due to a difference in the relative orientation of the two MMBL domains. In contrast, the relative orientation of the tandem MMBL domains of pyocin L1 matches those of the dimeric plant lectins very closely, with alignment of pyocin L1 with the snowdrop lectin homodimer (pdb ID: 1MSA) giving an rmsd of 4.81 Å. Comparison of the respective N- and C- terminal domains from pyocin L1 and putidacin L1 shows they possess very similar folds with rmsds of 2.77 Å and 2.02 Å, respectively (Figures 4C–D). The higher value for comparison of the N-terminal domains is due to the presence of a 2-strand extension to β-sheet two of the putidacin L1 N-terminal MMBL domain, which is absent from pyocin L1 and other MMBLs. In order to identify protein structures which share a similar fold to pyocin L1 we submitted the structure of the DALI server (http://ekhidna.biocenter.helsinki.fi/dali_server/start). The DALI server searches the protein data bank (PDB) to identify proteins structurally related to the query structure [41]. Significant structural homology was only identified for putidacin L1 and other proteins previously characterised as containing a MMBL fold such as the snowdrop lectin. MMBL dimers of plant origin often form higher order structures, however small angle X-ray scattering of pyocin L1 showed it to be monomeric in solution (Figure S4).

**Table 1:** Crystallographic data collection and refinement statistics.

	Sugar Free Form	D-Rhamnose Soak	D-Mannose Soak
**Data collection** a			
Space group	*C222_1_*	*C222_1_*	*C222_1_*
Cell dimensions, *a*, *b*, *c* (Å)	53.41, 158.40, 147.67	52.99, 160.65, 150.57	53.42, 162.1, 152.5
Resolution (Å)	36.42 - 2.09 (2.14 - 2.09)	54.99 - 2.37 (2.43 - 2.37)	55.53 -2.55 (2.67 - 2.55)
Solvent content (%)	56	55	56
No. of unique observations	37131 (2751)	26242 (1922)	22096 (2901)
Multiplicity	4.8 (4.9)	4.4 (4.5)	5.5 (5.7)
Completeness (%)	99.0 (99.8)	99.1 (99.5)	99.9 (100.0)
R_merge_ (%)	7.2 (59.2)	5.9 (83.0)	7.1 (85.6)
R_pim_ (%)^b^	4.1 (33.0)	3.4 (44.9)	3.3 (39.2)
Mean *I*/sigma (*I*)	14.3 (2.1)	19.0 (2.1)	13.3 (2.3)
**Refinement statistics**			
R_work_/R_free_ (%)	17.8/22.2	20.9/25.7	19.4/24.8
No. of non-hydrogen atoms	4505	4178	4138
RMSD of bond lengths (Å)	0.02	0.015	0.013
RMSD of bond angles (°)	1.96	1.63	1.70
No. of waters	344	95	27
Mean/Wilson plot B-value (Å^2^)	40.2/33.8	54.2/43.6	65.9/59.1
Ramachandran plot (%)^c^			
*Favoured/Allowed/Outliers*	97.2/2.2/0.6	97.4/2.2/0.4	96.6/3.0/0.4
PDB identifier	4LE7	4LED	4LEA

a Values in parentheses refer to the highest resolution shell.

b *R*_pim_ = Σ*_hkl_*[1/(N−1)]^1/2^Σ*_i_*|*I_i_*(*hkl*)−<*I*(*hkl*)>|/Σ*_hkl_*Σ*_i_I_i_*(*hkl*).

c Percentages of residues in favored/allowed regions calculated by the program RAMPAGE [68].

**Figure 5:**
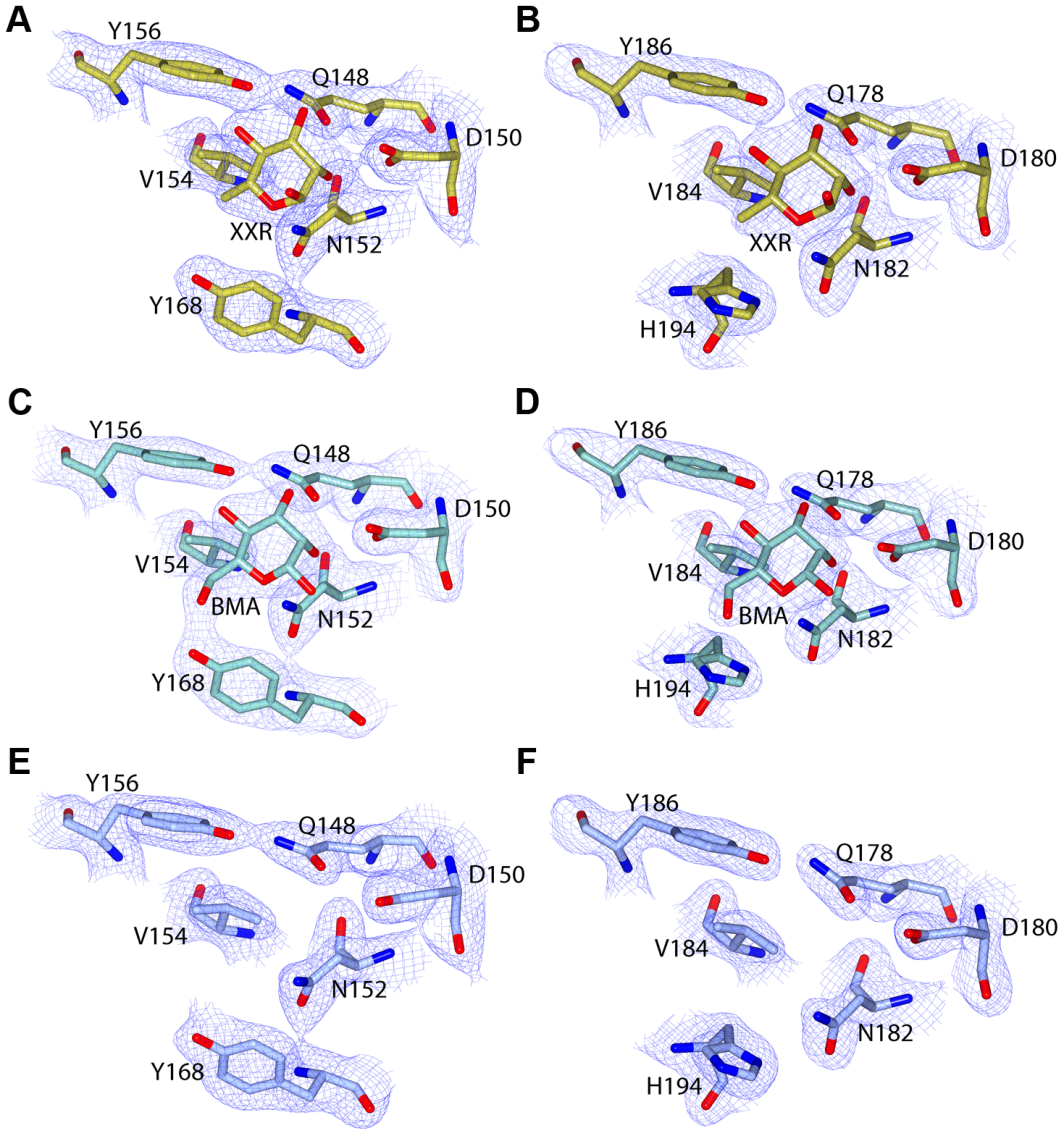
C-terminal MMBL-sugar binding motifs of pyocin L1 bind d-rhamnose and d-mannose. Electron density (at 1.3 σ) with fitted stick model of pyocin L1 MMBL-sugar binding site C1 with: (A) d-rhamnose (XXR), (C) d-mannose (BMA), (E) no bound sugar, and sugar binding site C2 with: (B) d-rhamnose, (D) d-mannose, (F) no bound sugar. For clarity, electron density is clipped to within 1.5 Å of visible atoms.

**Figure 6:**
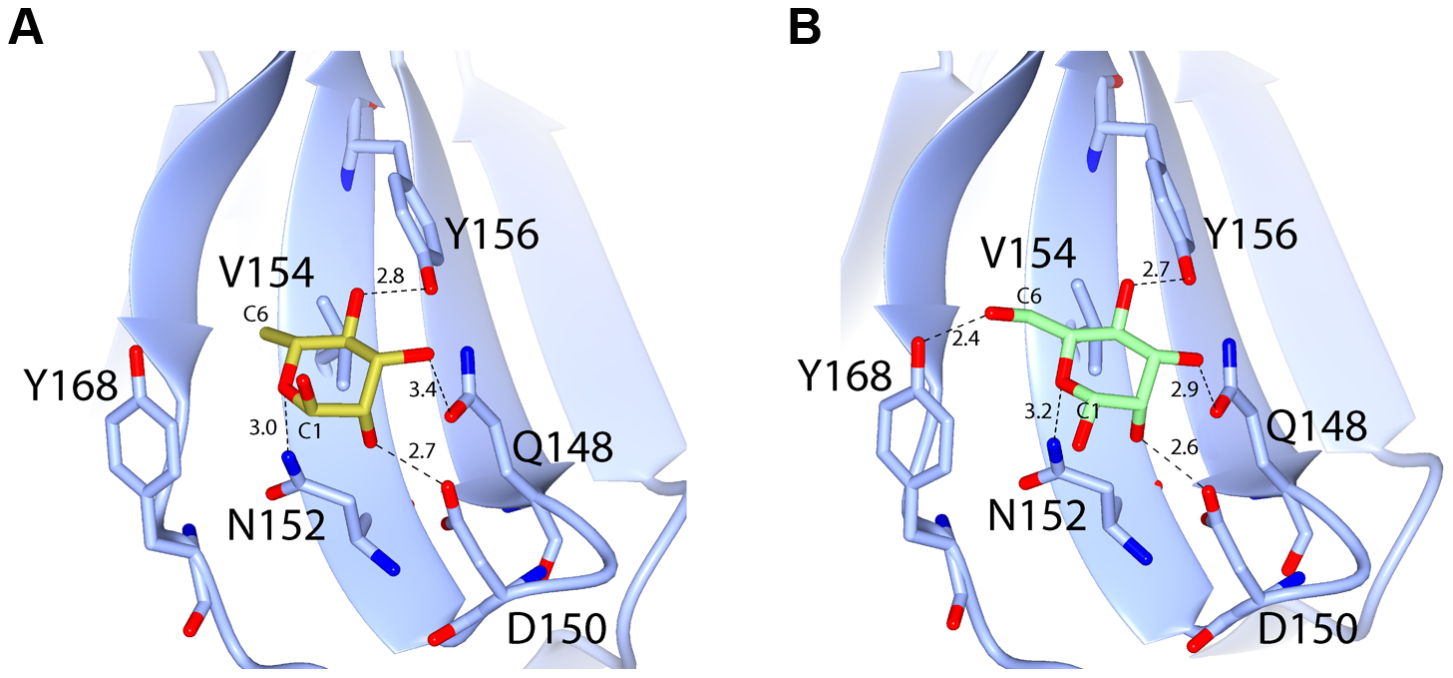
Hydrogen-bonding interactions between pyocin L1 MMBL sugar-binding motif C1 with d-rhamnose and d-mannose. Hydrogen bonds between protein side chains with (A) d-rhamnose and (B) d-mannose are shown; all distances are in Å.

Electron density maps, derived from both d-mannose and d-rhamnose soaked crystals show clear density for sugar moieties in both sites, C1 and C2 (Figure 5). The sugars refined well in these densities at full occupancy, giving B-factors comparable to the surrounding protein side chains. The canonical MMBL hydrogen bonds observed for both d-mannose and d-rhamnose were the same: Gln to O3, Asp to O2, Asn to O2 and Tyr to O4. In addition, O6 of d-mannose forms a hydrogen bond with Tyr169 in C1 and His194 in C2. As d-rhamnose is C6 deoxy d-mannose, it lacks these interactions (Figure 6). The fact that d-mannose forms an additional hydrogen bond is counter-intuitive given that pyocin L1 has a significantly stronger affinity for d-rhamnose, however Val154, Val163 and Ala166 of C1 and Val184 and Ala191 of C2 form a hydrophobic pocket to accommodate the C6-methyl group of d-rhamnose (Figure S5).

Weak density was observed for both sugars at site N1, however given the high concentrations used in the soak and the overall low binding affinity of pyocin L1 for monomeric sugars, it is unlikely that N1 represents a primary binding site for d-rhamnose (Figure S5). The conserved residues in site N2 form interactions with the C-terminal extension of the protein and as such are inaccessible. Weak density was also observed adjacent to the binding site C1 of mol B in both the soaks and in mol A of the d-rhamnose form. This density may correspond to a peripheral binding site utilised in binding to the carbohydrate chain of LPS, as is observed in the structure of putidacin L1 bound to oligosaccharides [23].

**Figure 7:**
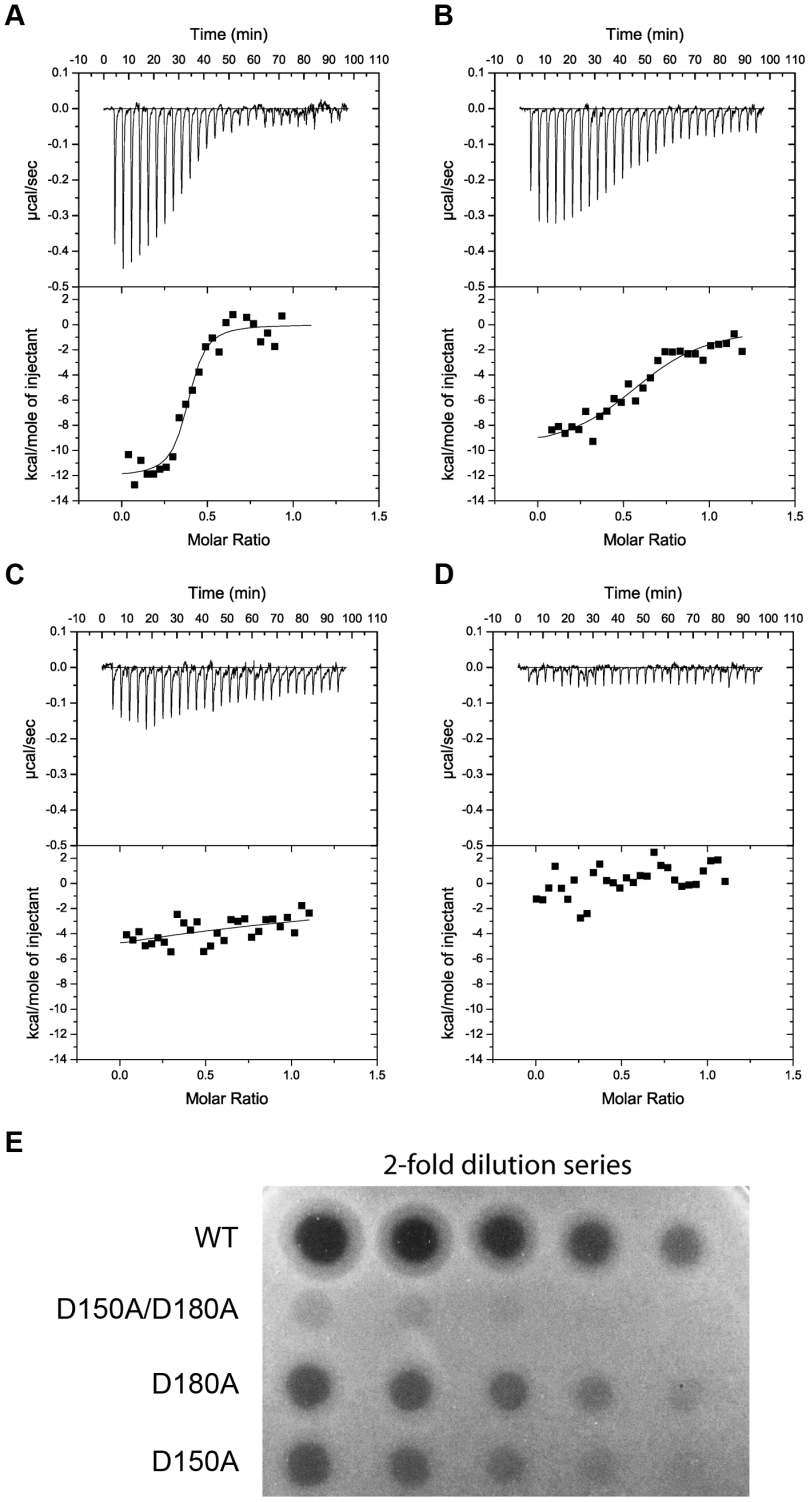
Binding of the CPA at the C-terminal sugar binding motifs, C1 and C2, is critical to pyocin L1 cytotoxicity. ITC binding isotherms of (A) wild-type (B) D180A (C) D150A and (D) D150A/D180A pyocin L1 all at (100 µM) titrated into isolated LPS-derived polysaccharide (1 mg ml^−1^) from wild-type *P. aeruginosa* PAO1. Fit to a single binding site model is shown. (E) Spot tests to determine cytotoxic activity of wild-type and pyocin L1 variants against of *P. aeruginosa* PAO1. Purified protein (starting concentration 400 µg ml^−1^ with 2-fold sequential dilutions) was spotted onto a growing lawn of *P. aeruginosa* PAO1. Clear zones indicate pyocin L1 cytotoxicity.

To test the idea that the observed binding of d-rhamnose to sites C1 and C2 is reflective of CPA binding and that this binding is critical to pyocin L1 cytotoxicity, we created pyocin L1 variants in which the conserved aspartic acids of the QxDxNxVxY motifs of the C1 and C2 sugar binding sites were mutated to alanine and compared their cytotoxicity and ability to bind the CPA by ITC with the wild-type protein. Titrations with wild-type pyocin L1 and the D150A (C1) and D180A (C2) variants were performed by titrating protein at a concentration of 100 µM into a solution of LPS-derived polysaccharide (1 mg ml^−1^) from strain PAO1 (Figure 7). Under these conditions we were able to generate binding isotherms that enabled us to accurately determine an apparent K_d_ of 0.15 (±0.07) µM for the wild-type pyocin L1-CPA complex. For both the D150A (C1) and D180A (C2) variants, affinity for CPA was reduced. For the pyocin L1 D150A-CPA complex a K_d_ of 1.52 (±0.51) µM was determined, a 10-fold increase in K_d_ relative to the wild-type pyocin L1-CPA complex. However, CPA binding to the D180A variant was severely weakened and although heats of binding were still observed the K_d_ for this complex, which could not be accurately determined, is likely >500 µM. We also produced a double mutant in which both D150A and D180A mutations were present. For this double mutant, no binding to CPA was observed by ITC. These data show that both the C1 and C2 sugar binding motifs are required for full CPA binding, but that the C2 binding site is the major CPA binding determinant. The killing activity of these sugar binding motif variants showed a good correlation with their ability to bind the CPA. Both the D150A and D180A variants showed reduced cytotoxicity against PAO1 relative to pyocin L1, with the D150A showing a greater reduction in activity and for the D150A/D180A variant very low levels of cytotoxicity were observed (Figure 7).

### Putidacin L1 binds to *P. syringae* LPS and d-rhamnose

Pyocin L1 targets sensitive strains of *P. aeruginosa* through binding to LPS and utilises this as a cell surface receptor. To determine if LPS binding is common to the homologous and previously characterised lectin-like bacteriocin putidacin L1, we purified this protein and determined if the susceptibility of a number of strains of *P. syringae* correlated with the ability of putidacin L1 to bind to LPS-derived carbohydrates from these strains.

**Figure 8:**
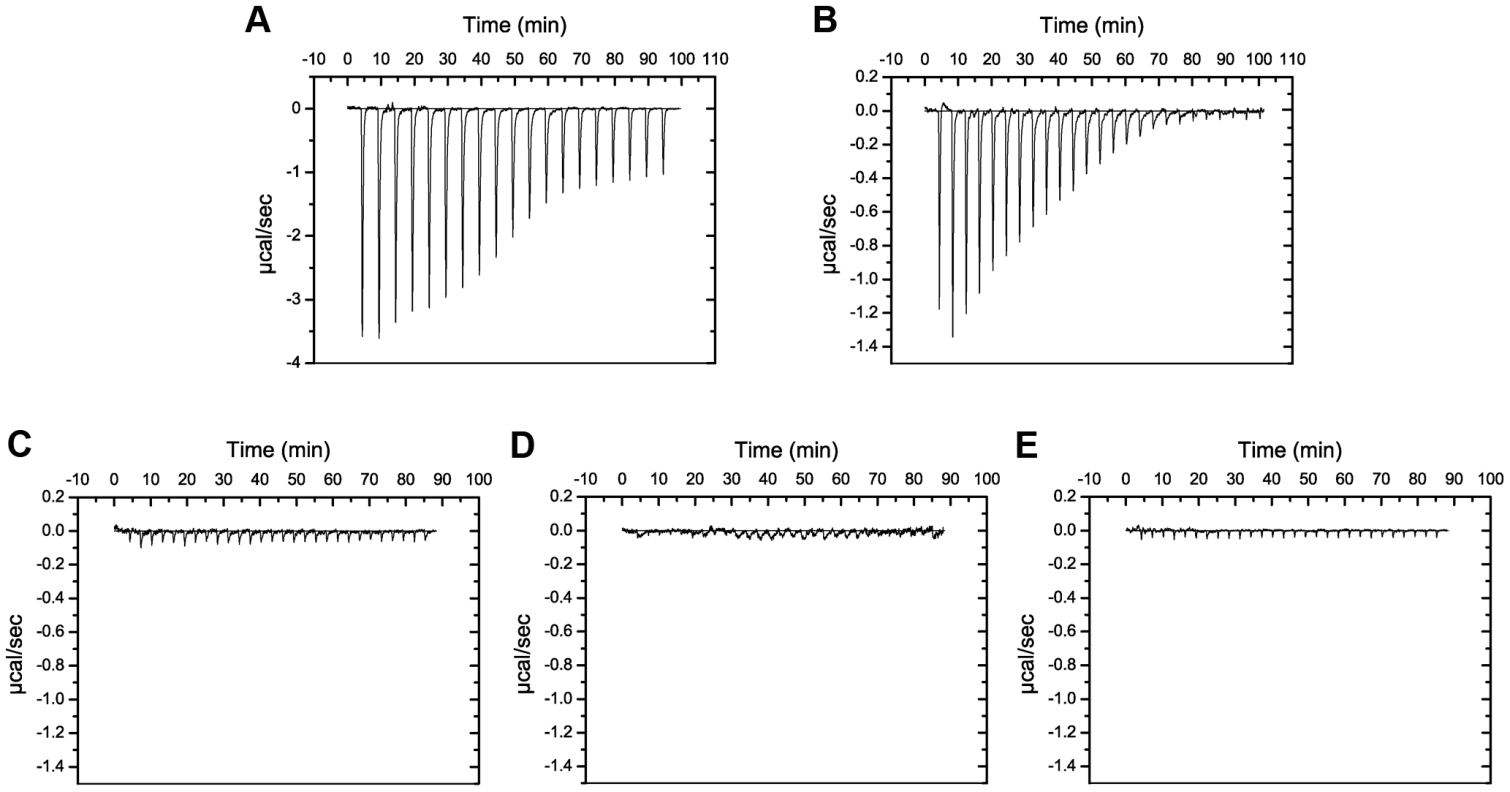
Putidacin L1 binds strongly to LPS-derived polysaccharides from susceptible but not tolerant or resistant *P. syringae* isolates. ITC isotherm of LPS-derived polysaccharides (3 mg ml^−1^) from strains highly sensitive to putdacin L1: (A) *P. syringae* LMG 2222, (B) *P. syringae* LMG 5456 titrated into putidacin L1 (60 µM). Large, saturable heats are indicative of binding. LPS-derived polysaccharides (3 mg ml^−1^) from strains non-sensitive to putidacin L1: (C) *P. syringae* NCPPB 2563, (D) *P. syringae* DC3000, or highly tolerant (E) *P. syringae* LMG 1247 to putidacin L1, show no heats of binding when titrated into putidacin L1 (60 µM).

From the five strains of *P. syringae* tested, LMG 5456 and LMG 2222 were found to be highly susceptible to putidacin L1 with killing down to concentrations of 0.3 and 7.6 nM respectively. DC3000 and NCPPB 2563 showed complete resistance and LMG 1247 was highly tolerant (killing down to 0.6 µM). Binding of putidacin L1 to the isolated LPS-derived polysaccharides of the above mentioned strains was tested by ITC. Large saturable heats of binding were observed for putidacin L1 and the LPS-derived polysaccharides from LMG 5456 and LMG 2222, while no binding was observed between putidacin L1 and the LPS-derived polysaccharides from LMG 1247, 2563 or DC3000 (Figure 8). Thus, there is excellent correlation between putidacin L1 cell killing and the binding of LPS-derived polysaccharide indicating that like pyocin L1, putidacin L1 utilises LPS as a surface receptor.

Although *P. syringae* O-antigens are diverse relative to CPA, the incorporation of d-rhamnose is widespread and seemingly almost universal in strains of this species [42], [43]. Interestingly, in cases where d-rhamnose is not a component of *P. syringae* LPS, l-rhamnose is present [42]. As with pyocin L1 we utilised ITC and NMR to characterise the binding affinity of putidacin L1 for d-rhamnose, in comparison with d-mannose and l-rhamnose. Putidacin L1 exhibited an affinity of 5–10 mM for d-rhamnose, which is comparable to that of pyocin L1, and approximately 10-fold stronger than its affinity for d-mannose (Figure S6). Interestingly, no binding of l-rhamnose to putidacin L1 or pyocin L1 was observed (Figure S7). It is interesting to note that in the strains of *P. syringae* we have tested, the killing spectrum (but not the potency) of pyocin L1 and putidacin L1 is identical. This observation combined with the specificity of putidacin L1 for d-rhamnose, strongly suggests that it also binds to a d-rhamnose containing O-antigen. Indeed branched d-rhamnose O-antigens are common in *P. syringae* [42], [43].

Our data for both pyocin L1 and putidacin L1 indicate that d-rhamnose containing O-antigens are utilised as surface receptors for lectin-like bacteriocins from *Pseudomonas* spp. This is an attractive hypothesis since the inclusion of d-rhamnose in the lipopolysaccharides from members of this genus is widespread and could form an important component of the genus specific activity of this group of bacteriocins.

## Discussion

In this work we have shown that pyocin L1 targets susceptible cells through binding to the CPA component of LPS and that primary recognition of CPA occurs through binding of d-rhamnose at the conserved QxDxNxVxY sugar binding motifs of the C-terminal lectin domain. The ability of both pyocin L1 and putidacin L1 to recognise d-rhamnose containing carbohydrates is an important component of their ability to target sensitive strains of *Pseudomonas* spp. The use of the O-antigen as a primary receptor differentiates the lectin-like bacteriocins from other multidomain bacteriocins such as colicins and S-type pyocins (colicin-like bacteriocins) which utilise outer membrane proteins as their primary cell surface receptors [44]. The colicin-like bacteriocins also possess a flexible, or natively disordered N-terminal region that is thought to pass through the lumen of a coreceptor and interact with the periplasmic Tol or Ton complexes that mediate translocation of the bacteriocin across the outer membrane [11], [44]. The lack of such a flexible N-terminal region in the lectin-like bacteriocins suggests that either they do not need to cross the outer membrane in order to mediate their cytotoxicity or they do so by a mechanism that is fundamentally different to the diverse family of colicin-like bacteriocins. Given the extensive structural homology between the lectin-like bacteriocins and plant lectins it seems likely that these bacteriocins share a common ancestor with plant lectins and from an evolutionary perspective are unrelated to the colicin-like bacteriocins.

In addition to O-antigen recognition, additional factors, as yet to be determined, are clearly also important in strain and species specificity among the lectin-like bacteriocins. Indeed, recent work from Ghequire *et al.* has shown through domain swapping experiments that for putidacin L1 (LlpA_BW_) and the homologous lectin-like bacteriocin LlpA1_Pf-5_ from *Pseudomonas fluorescens*, species specificity is governed by the identity of the N-terminal lectin domain [23]. Thus, in view of these data and our own data it seems likely that the C-terminal lectin domain of this class of bacteriocins plays a general role in the recognition of d-rhamnose containing O-antigens, with the N-terminal domain interacting with species-specific factors and thus determining the precise species and strain specificity of these bacteriocins. Although there are few clues as to how the lectin-like bacteriocins ultimately kill susceptible cells, we have established a clear role for the C-terminal MMBL domain of these proteins. The roles of the N-terminal MMBL domain and the C-terminal extension remain to be discovered [23]. However, from the previous work of Ghequire *et al*, it is clear that all three of these regions are required for killing of susceptible cells.

Interestingly, although rhamnose is frequently a component of plant and bacterial glycoconjugates, such as the rhamnolipids of *P. aeruginosa* [45] and pectic polysaccharides of plant cell walls [46], it is generally the l-form of this sugar that is found in nature. Although otherwise rare, d-rhamnose is found frequently as a component of the LPS of plant pathogens and plant associated bacteria such as *P. syringae* [42], [43], *P. putida* [47], *Xanthomonas campestris* [48] and *Burkholderia* spp. [49], but is a relatively rare component of the O-antigens of animal pathogens such as *E. coli*, *Salmonella* and *Klebsiella*. It is interesting to speculate that since d-rhamnose is a common component of the LPS of bacterial plant pathogens, that some of the many lectins produced by plants may have evolved to target d-rhamnose as part of plant defence to bacterial pathogens.

The specificity of lectin-like bacteriocins suggests that these protein antibiotics may be useful in combating plant pathogenic bacteria, either through the use of bacteriocin expressing biocontrol strains or by the production of transgenic plants engineered to express these proteins. The specific targeting mechanism described here, binding of d-rhamnose containing polymers, indicates that the lectin-like bacteriocins would not interact with either plant or animal cells, since these lack d-rhamnose containing glycoconjugates. In addition, these narrow spectrum antibiotics would leave the majority of the soil microbiome and the gut microbiome of plant-eating animals intact and so would be likely to have minimal environmental impact and minimal impact on animal health. This latter property and the potency of these protein antibiotics could also make the use of lectin-like bacteriocins in the treatment of chronic multidrug-resistant *P. aeruginosa* infections in humans an attractive proposition.

## Materials and Methods

### Bacterial strains, plasmids and growth conditions

Strains and plasmids utilised in this study are presented in Supplementary Table S1. Strains of *P. aeruginosa* were grown in LB at 37°C, *P. syringae* were grown in King's B Media (KB) (20 g peptone, 10 g glycerol, 1.5 g MgSO_4_, 1.5 g K_2_HPO_4_ per liter adjusted to pH 7.5) at 28°C.

### Cloning and purification of lectin-like bacteriocins

Pyocin L1 was amplified from the genomic DNA of the producing strain *P. aeruginosa* C1433 [50] by PCR using primers designed to introduce an NdeI site at the start of the *pyoL1* gene (ACA GAT CAT ATG AAG TCT CCA AAC AAA AGG AGG) and an XhoI site at the end of the gene (ACA GAT CTC GAG GAC CAC GGC GCG CCG TCG TGG ATA GTC GTG GGG CCA A). The PCR product was ligated into the corresponding sites of the *E. coli* expression vector pET21a to give pETPyoL1 which encodes pyocin L1 with a C-terminal His_6_ tag separated from the C-terminus of pyocin L1 by a 6 amino acid linker (RRRAVV). Pyocin L1 was overexpressed from *E. coli* BL21(DE3)pLysS carrying the plasmid pETPyoL1. Five litres of LB broth was inoculated (1∶100) from an overnight culture and cells were grown at 37°C in a shaking incubator to an OD_600_ = 0.6. Protein production was induced by the addition of 0.3 mM isopropyl β-d-1-thiogalactopyranoside (IPTG), the cells were grown at 22°C for a further 20 hand harvested by centrifugation. Cells were resuspended in 20 mM Tris-HCl, 500 mM NaCl, 5 mM imidazole (pH 7.5) and lysed using an MSE Soniprep 150 (Wolf Laboratories) and the cell debris was separated by centrifugation. The cell-free lysate was applied to a 5-ml His Trap HP column (GE Healthcare) equilibrated in 20 mM Tris-HCl, 500 mM NaCl, 5 mM imidazole (pH 7.5) and pyocin L1 was eluted over a 5–500 mM imidazole gradient. Pyocin L1 containing fractions were identified by SDS PAGE, pooled and dialyzed overnight into 50 mM Tris-HCl, 200 mM NaCl, pH 7.5 and remaining contaminants were removed by gel filtration chromatography on a Superdex S75 26/600 column (GE Healthcare) equilibrated in the same buffer. The protein was concentrated using a centrifugal concentrator (Vivaspin 20) with a molecular weight cut off of 5 kDa and stored at −80°C until required. The putidacin L1 open reading frame was synthesised (DNA 2.0) and cloned into pET21a via 5′ NdeI and 3′ XhoI restriction sites. The stop codon was removed in order to utilise the pET21a C-terminal His_6_ tag. Purification of putidacin L1 was performed as for pyocin L1. Constructs to express the pyocin L1 mutants D31A, D97A, D150A and D180A were created using the QuikChange Site Directed Mutagenesis Kit (Stratagene) utilising pETPyoL1 as a template. The primers used were CAA ATT GGT CAT GCA AGC GGC TGG CAA CTT GGT CCT TTA CG and CGT AAA GGA CCA AGT TGC CAG CCG CTT GCA TGA CCA ATT TG for D31A, GCG TAC CTG AAT CTT CAA GAT GCT GGG GAC TTC GGT ATA TTT TC and GAA AAT ATA CCG AAG TCC CCA GCA TCT TGA AGA TTC AGG TAC GC for D97A, CGC CTA GCG TTT CAG GGA GCT GGC AAC CTA GTG ATC TAT C and GAT AGA TCA CTA GGT TGC CAG CTC CCT GAA ACG CTA GGC G for D150A and GAT AGA GCA GTA GTG CAA GAG GCT GGA AAT TTT GTT ATC TAC AAA G and CTT TGT AGA TAA CAA AAT TTC CAG CCT CTT GCA CTA CTG CTC TAT C for D180A. Mutant proteins were purified as described above for wild-type pyocin L1.

### Pyocin sensitivity assays: Overlay spot plate method

Soft agar overlay spot plates were performed using the method of [35]. 150 µl of test strain culture at OD_600_ = 0.6 was added to 6 ml of 0.8% soft agar and poured over an LB or KB agar plate. 5 µl of bacteriocin at varying concentrations was spotted onto the plates and incubated for 20 h at 37 or 28°C.

### Isolation of pyocin L1 tolerant mutants

1.5 ml of a culture of *P. aeruginosa* E2 (OD_600_ = 0.6) was centrifuged and resuspended in 100 µl of LB, to which 100 µl (8 mg ml^−1^) of purified pyocin L1 was added. The culture was grown for 1 h, plated onto a LB agar plate and incubated for 20 h at 37°C. Isolated colonies were identified as *P. aeruginosa* using 16S PCR as described previously [51].

### Whole genome sequencing

The genomes of *P. aeruginosa* E2 and derived pyocin L1 tolerant mutants were sequenced at the Glasgow Polyomics Facility, generating paired-end reads on an Illumina MiSeq Personal Sequencer. Reads were mapped to the previously sequenced parent genomes of *P. aeruginosa* E2 using the CLC genomics workbench, MAUVE and RAST to create an ordered annotated genome. The CLC genomics workbench was used for genome comparisons and the identification of SNPs/INDELs.

### LPS purification and isolation of LPS-derived polysaccharide

LPS was purified from 1 litre cultures of *P. aeruginosa* and *P. syringae* strains as described previously, with modifications including the omission of the final trifluoroacetic acid hydrolysis and chromatography steps [52]. Cells were grown for 20 h at 37°C and 28°C for *P. aeruginosa* and *P. syringae* respectively, pelleted by centrifugation at 6000 g for 20 min, and resuspended in 50 mM Tris, pH 7.5 containing lysozyme (2 mg ml^−1^) and DNase I (0.5 mg ml^−1^). Cells were lysed by sonication and the cell lysate was incubated at 20°C for 30 min before EDTA was added to a final concentration of 2 mM. An equal volume of aqueous phenol was added and the solution was heated at 70°C for 20 min, with vigorous mixing. The solution was then cooled on ice for 30 min, centrifuged at 7000 g for 20 min and the aqueous phase extracted. Proteinase K was added to a final concentration of 0.05 mg ml^−1^ and dialysed for 12 h against 2×5 L H_2_O. LPS was pelleted by ultracentrifugation at 100,000 g for 1 h, resuspended in H_2_O and heated to 60°C for 30 min to remove residual proteinase K activity. LPS-derived carbohydrates were isolated by heating LPS in 2% acetic acid for 1.5 h at 96°C. Lipid A was removed by centrifugation at 13,500 g for 3 min followed by extraction with an equal volume of chloroform. The aqueous phase was then lyophilised.

### SDS-PAGE, silver staining and immunoblotting

Purified LPS from wild-type and mutant samples were resolved by electrophoresis on 12% SDS-polyacrylamide gels. The LPS banding patterns were visualised by the Invitrogen ultrafast silver staining method. For immunoblotting LPS was transferred onto nitrocellulose membranes and western immunoblotting was performed as previously described using the CPA-specific monoclonal antibody N1F10 and alkaline phosphatase-conjugated goat anti-mouse Fab2 as the secondary antibody [39]. The blots were developed using SIGMAFAS BCIP/NBT tablets.

### Isothermal titration calorimetry

ITC experiments were performed on a VP-ITC microcalorimeter (MicroCal LLC). For monosaccharide binding, titrations were carried out at 299 K with regular 15 µl injections of ligands into 60–100 µM pyocin L1 or putidacin L1 at 300 s intervals. 50 mM d-rhamnose, d-mannose or l-rhamnose were used as titrants and reactions were performed in 0.2 M sodium phosphate buffer, pH 7.5. d-rhamnose (>97%) was obtained from Carbosynth Limited (UK) and d-mannose and l-rhamnose (>99%) from Sigma-Aldrich (UK). For O-antigen-pyocin L1 binding reactions, pyocin L1 or pyocin L1 variants were used as titrant at 100 or 150 µM with cleaved O-antigen sugars dissolved at 1 mg ml^−1^ in the chamber. For curve fitting we estimated the molar concentration of LPS-derived CPA containing carbohydrate chains at 20 µM based on an estimated average molecular weight of 10 kDa for CPA containing polysaccharides and estimating the percentage of total LPS represented by CPA containing carbohydrates as 20% of the total by weight [53]. This value may not be accurate and as such the stoichiometry implied by the fit is likely to be unreliable. However, the use of this estimated value has no impact on the reported parameters of ΔH, ΔS and K_d_. For O-antigen-putidacin L1 binding reactions, O-antigen was used as the titrant at 3 mg ml^−1^ with 60 µM putidacin L1 in the chamber. Reactions were performed in 20 mM HEPES buffer pH 7.5. All samples were degassed extensively prior to the experiments. Calorimetric data were calculated by integrating the area under each peak and fitted with a single-site binding model with Microcal LLC Origin software. The heats of dilution for each titration were obtained and subtracted from the raw data.

### NMR titration experiments

NMR chemical shift perturbation analysis of sugar binding by pyocin L1 and putidacin L1 was carried out at 305 K and 300 K respectively. Fast-HSQC spectra [54] were recorded using ^15^N labelled proteins (0.1–0.2 mM) and unlabelled ligands, d-rhamnose and d-mannose (100 mM), on a Bruker AVANCE 600 MHz spectrometer. Protein samples were prepared with and without the sugars present and volumes were exchanged at fixed ratios, making sure the protein concentration remained unchanged. The spectra were processed with Topspin and analysed with CCPNmr analysis [55].

### Crystallisation and data collection for pyocin L1

Purified pyocin L1 at a concentration of 15 mg ml^−1^ was screened for crystallisation conditions using the Morpheus and PGA crystallisation screens (Molecular Dimensions) [56]. Screens were prepared using a Cartesian Honeybee 8+1 dispensing robot, into 96-well, MRC-format, sitting drop plates (reservoir volume of 80 µl; drop size of 0.5 µl of protein and 0.5 µl of reservoir solution). Clusters of needle shaped crystals grew in a number of conditions in each screen over 3 to 7 days. Two of these conditions, condition 1 (20% v/v ethylene glycol, 10% w/v PEG 8000, 0.03 M CaCl_2_, 0.03 M MgCl_2_, 0.1 M Tris/Bicine, pH 8.5) and condition 2 (20% PEG 550 MME, 20% PEG 20 K, 0.03 M CaCl_2_, 0.03 M MgCl_2_ 0.1 M MOPS/HEPES, pH 7.5) from the Morpheus screen were selected for optimisation by vapour diffusion in 24 well plates (reservoir volume 500 µl, drop size 1 µl protein and 1 µl reservoir solution). Clusters of needles from these trays grew after 3–7 days and were mechanically separated. The un-soaked crystals were from condition 1, while soaked crystals were from condition 2. Un-soaked crystals were looped and directly cryo-cooled to 110 K in liquid nitrogen; D-mannose and D-rhamnose soaked crystals were soaked for 2–12 min in artificial mother liquor containing 4 M d-mannose or 2 M d-rhamnose, before cryo-cooling to 110 K. X-ray diffraction data were collected at the Diamond Light Source, Oxfordshire, UK at beam lines I04, I04-1 and I24. Automatic data processing was performed with Xia2 within the EDNA package [57].

### Structure solution and refinement for pyocin L1

A dataset from an un-soaked pyocin L1 crystal was submitted to the Balbes pipeline along with the amino acid sequence for pyocin L1 [58]. Balbes produced a partial molecular replacement solution based on the structure of *Galanthus nivalis* agglutinin (PDB ID: 1MSA). Initial phases from Balbes were improved via density modification and an initial model was built using Phase and Build from the Phenix package [59]. The model was then built and refined using REFMAC5 and Coot 0.7 [60], [61]. Validation of all models was performed using the Molprobity web server and Procheck from CCP4-I [62], [63]. Two structures of sugar soaked pyocin L1 were solved by molecular replacement using Phaser [64], with the sugar-free pyocin L1 as the search model. Additional electron density corresponding to bound sugars, was observed in both 2*F*_o_-2*F*_c_ and *F*_o_-*F*_c_ maps [65]. Sugars were fitted and structures refined using Coot 0.7 and REFMAC5. β-d-mannose (PDB ID: BMA) corresponded best to the density of bound d-mannose. The density in the d-rhamnose complex best corresponded to α-d-rhamnose, for which no PDB ligand exists; a model for α-d-rhamnose was prepared by removing the oxygen from carbon 6 of α-d-mannose and submitting these PDB coordinates to the Prodrg server, which generated the model and modeling restraints [65]. The resultant α-d-rhamnose was designated with the PDB ID: XXR.

### Small angle X-ray scattering

SAXS was carried out on the X33 beamline at the Deutsches Elektronen Synchrotron (DESY, Hamburg, Germany). Data were collected on samples of Pyocin L1 in the range of 0.5–5 mg ml^−1^. Buffer was read before and after each sample and an average of the buffer scattering was subtracted from the sample scattering. The data obtained for each sample were analysed using PRIMUS [66], merging scattering data at low angles with high angle data. The distance distribution function, p(r), was obtained by indirect Fourier transform of the scattering intensity using GNOM [67]. A Guinier plot (ln I(s) vs s^2^) was used to calculate the molecular weight at I(0) and radius of gyration, R_g_, of PyoL1. *Ab initio* models of the protein in solution were built using DAMMIF [68], averaged with DAMAVER [69] and overlaid with the available crystal structure using SUPCOMB [70].

## Supporting Information

Figure S1 **Sequence alignment of pyocin L1 and previously reported MMBL-like bacteriocins.** Dark blue shading designates sequence identity, light blue designates chemically conserved residues. The three conserved MMBL sugar-binding motifs (N1, C1 and C2) and the partially conserved motif (N2) are boxed in red.
(JPG)

Figure S2 **Genetics of CPA biosynthesis in** ***P. aeruginosa*** **.** (A) CPA operon, annotated with location of *P. aeruginosa* E2 tolerant mutant (M4 and M11) deletion and PAO1 transposon insertion mutants. (B) Summary of CPA biosynthetic pathway, showing function performed by genes, shown to induce pyocin L1 tolerance or resistance.
(TIF)

Figure S3 **^1^H-^15^N HSQC spectra of ^15^N-labelled pyocin L1 in presence (red) and absence (black) of 100 mM (A) d-rhamnose and (B) d-mannose, showing distinctive chemical shifts upon addition of associating sugars.** Chemical shift changes specific to a small number of cross-peaks illustrates association of the sugars with a small subset of amino acids, which likely correspond to the residues within the binding sites. Analogous changes are observed for d-rhamnose and d-mannose titrations indicative that the same sites are binding both ligands. Greater shift magnitude is observed for d-rhamnose, indicative of a greater affinity towards this monosaccharide. Boxed regions include cross-peaks used for chemical shift perturbation analysis as shown in Figure 3.
(TIF)

Figure S4 **Small angle X-ray scattering of pyocin L1.** (A) *Ab initio* model of pyocin L1 computed with DAMMIF overlaid with the crystal structure. (B) Guinier plot of scattering data indicates that the protein is monomeric in solution (I(0) gives a molecular mass of 29.53 kDa) by extrapolation of scattering intensity to zero scattering angle. Radius of gyration is 2.72 nm, indicative of a folded, globular monomeric particle in solution.
(TIF)

Figure S5 **Coordination of d-rhamnose in C1, C2 and N2 binding sites of pyocin L1.** (A) Stereo view of d-rhamnose coordination by binding site C1 (A), C2 (C) and N1 (E), from d-rhamnose soak data. Core binding motif residues (blue) and additional residues contributing to the pocket (white) are shown. Omit map density for d-rhamnose in binding site C1 (B), C2 (D), N1 (F) calculated by refinement of data from d-rhamnose soaked crystal with model built from unsoaked crystal. Density for all sites contoured to 0.15e/Å^3^.
(TIF)

Figure S6 **Putidacin L1 shows specificity for d-rhamnose, compared with d-mannose.** (A) ITC isotherm of d-rhamnose (50 mM) titrated into putidacin L1 (0.1 mM). Weakly saturable heats are indicative of binding with modest affinity (Kd ∼5–10 mM). (B) ITC isotherm of d-mannose (50 mM) titrated into putidacin L1 (0.1 mM). Binding is undetectable under reaction conditions.
(TIF)

Figure S7 **Putidacin L1 and pyocin L1 do not bind l-rhamnose.** ITC isotherms of l-rhamnose (50 mM) titrated into putidacin L1 (A) and pyocin L1 (B) both at (0.1 mM). Binding is undetectable under these conditions.
(TIF)

Table S1 **Strains and plasmids used in this work.**
(PDF)

Text S1 **References for supplementary information.**
(DOCX)
