# Supplementary figures and images for: Lectin-Like Bacteriocins from Pseudomonas spp. Utilise D-Rhamnose Containing Lipopolysaccharide as a Cellular Receptor
Source: PLoS Pathog. 2014 Feb 6;10(2):e1003898. doi: 10.1371/journal.ppat.1003898 (PMC3916391; doi:10.1371/journal.ppat.1003898)

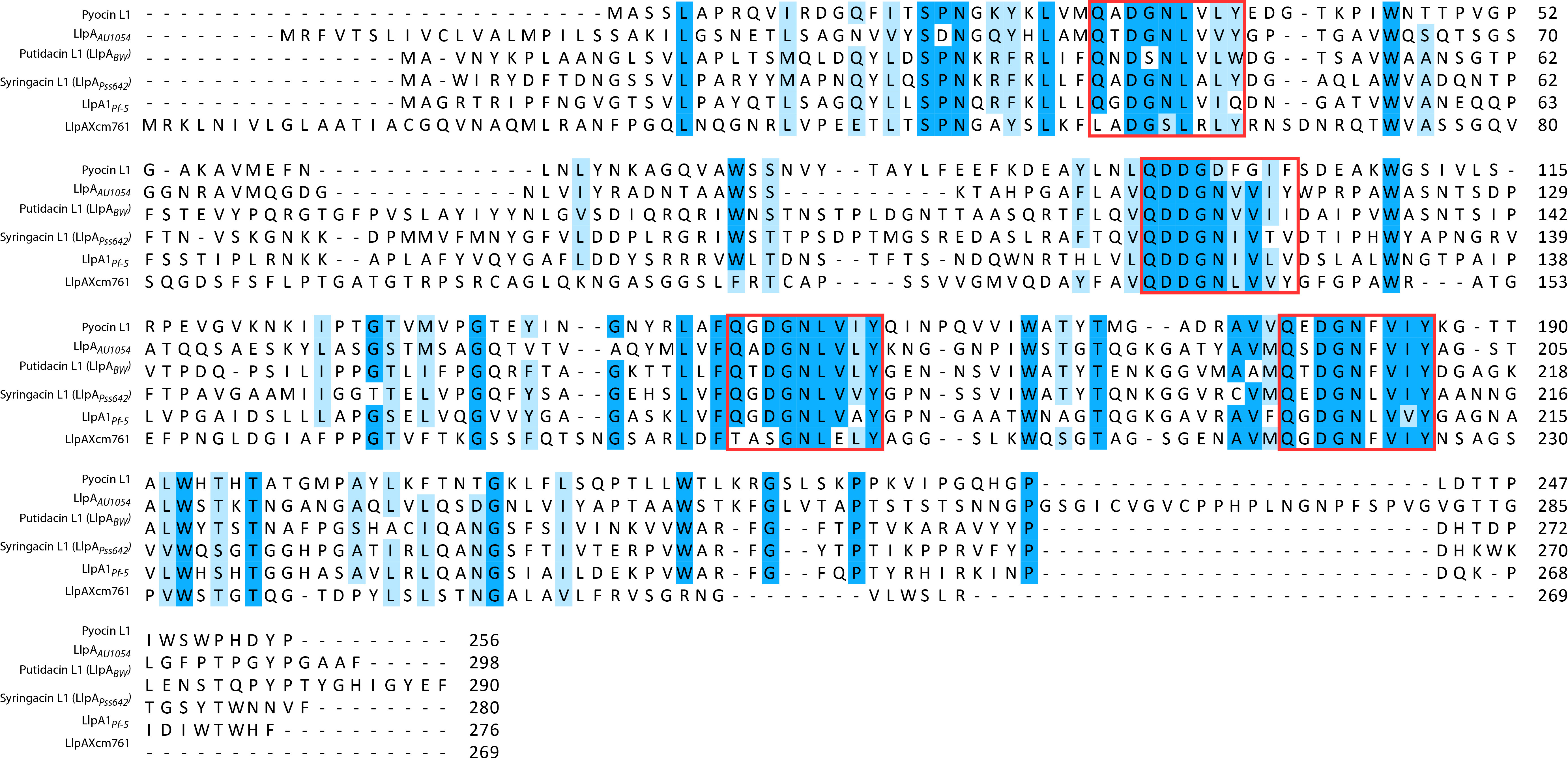

Supplement: Figure S1 — Sequence alignment of pyocin L1 and previously reported MMBL-like bacteriocins. Dark blue shading designates sequence identity, light blue designates chemically conserved residues. The three conserved MMBL sugar-binding motifs (N1, C1 and C2) and the partially conserved motif (N2) are boxed in red. (JPG) [file ppat.1003898.s001.jpg]

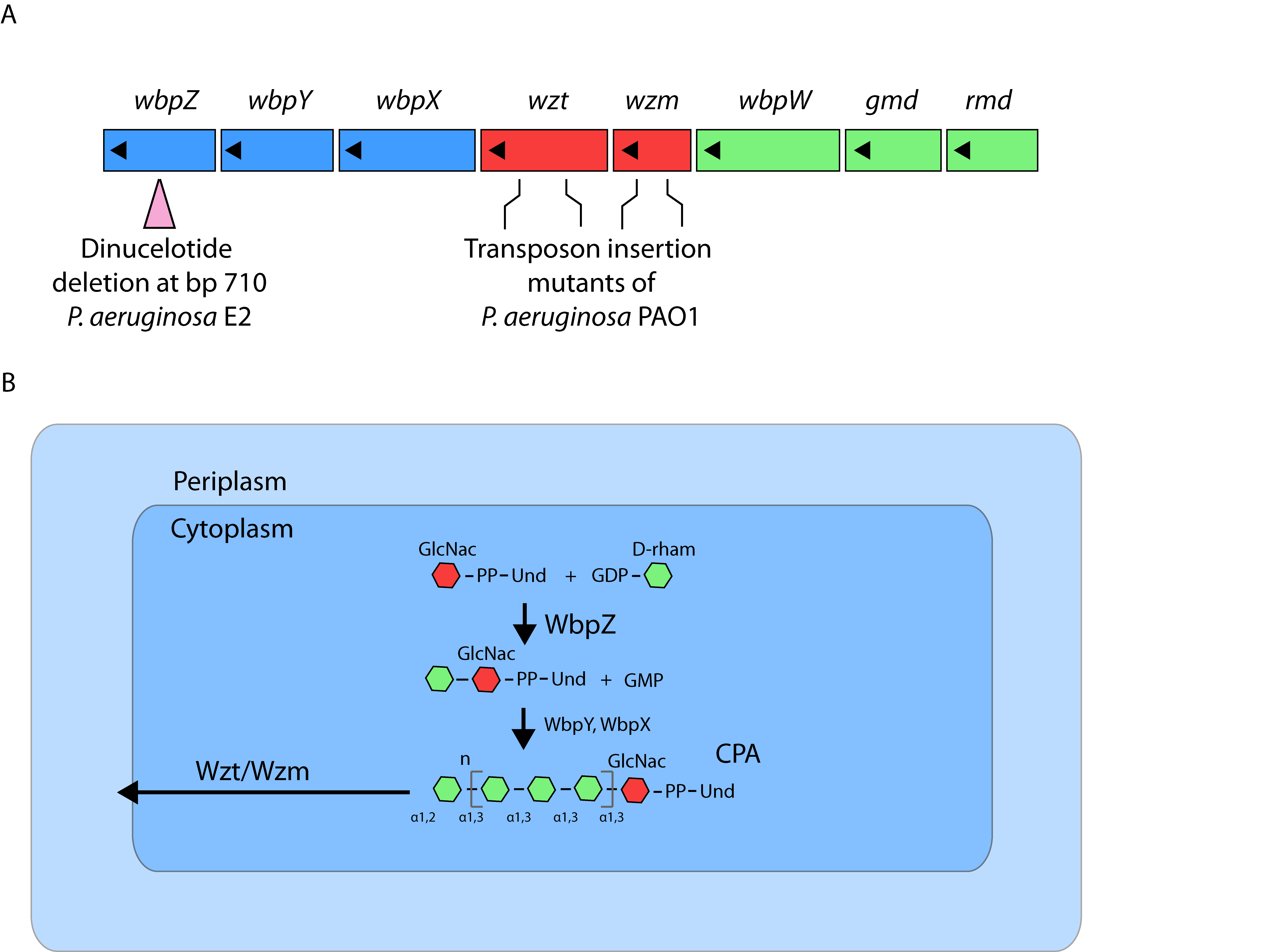

Supplement: Figure S2 — Genetics of CPA biosynthesis in P. aeruginosa . (A) CPA operon, annotated with location of P. aeruginosa E2 tolerant mutant (M4 and M11) deletion and PAO1 transposon insertion mutants. (B) Summary of CPA biosynthetic pathway, showing function performed by genes, shown to induce pyocin L1 tolerance or resistance. (TIF) [file ppat.1003898.s002.tif]

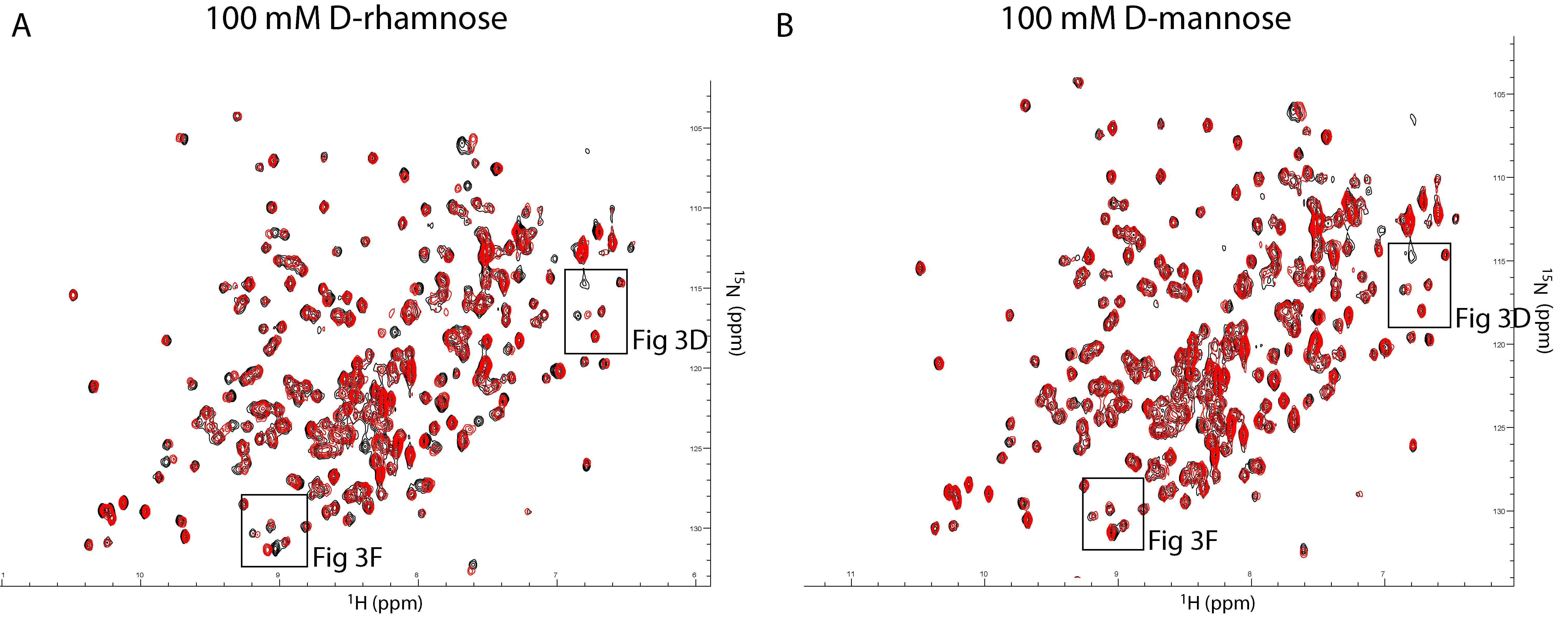

Supplement: Figure S3 — 1H-15N HSQC spectra of 15N-labelled pyocin L1 in presence (red) and absence (black) of 100 mM (A) d-rhamnose and (B) d-mannose, showing distinctive chemical shifts upon addition of associating sugars. Chemical shift changes specific to a small number of cross-peaks illustrates association of the sugars with a small subset of amino acids, which likely correspond to the residues within the binding sites. Analogous changes are observed for d-rhamnose and d-mannose titrations indicative that the same sites are binding both ligands. Greater shift magnitude is observed for d-rhamnose, indicative of a greater affinity towards this monosaccharide. Boxed regions include cross-peaks used for chemical shift perturbation analysis as shown in Figure 3. (TIF) [file ppat.1003898.s003.tif]

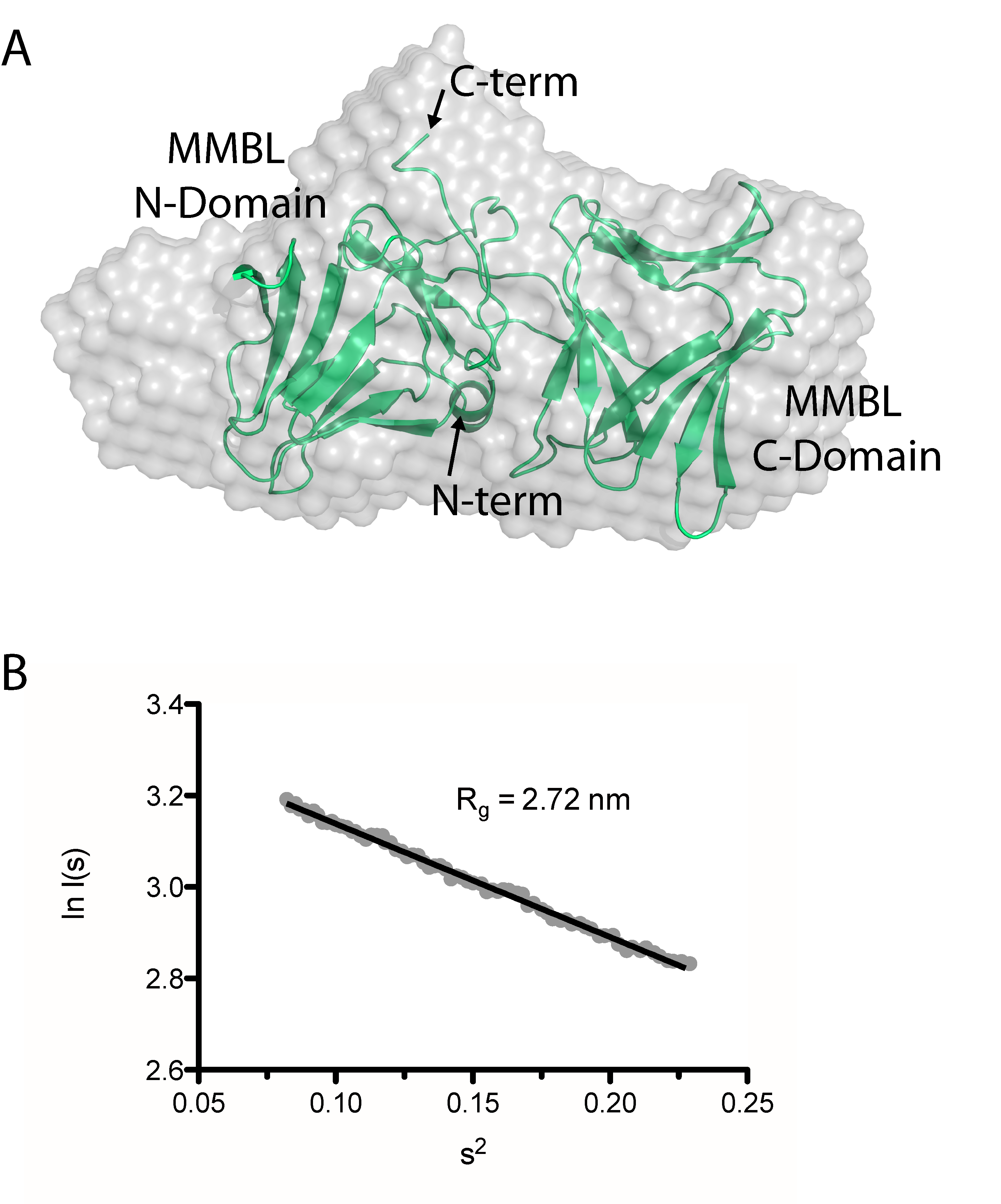

Supplement: Figure S4 — Small angle X-ray scattering of pyocin L1. (A) Ab initio model of pyocin L1 computed with DAMMIF overlaid with the crystal structure. (B) Guinier plot of scattering data indicates that the protein is monomeric in solution (I(0) gives a molecular mass of 29.53 kDa) by extrapolation of scattering intensity to zero scattering angle. Radius of gyration is 2.72 nm, indicative of a folded, globular monomeric particle in solution. (TIF) [file ppat.1003898.s004.tif]

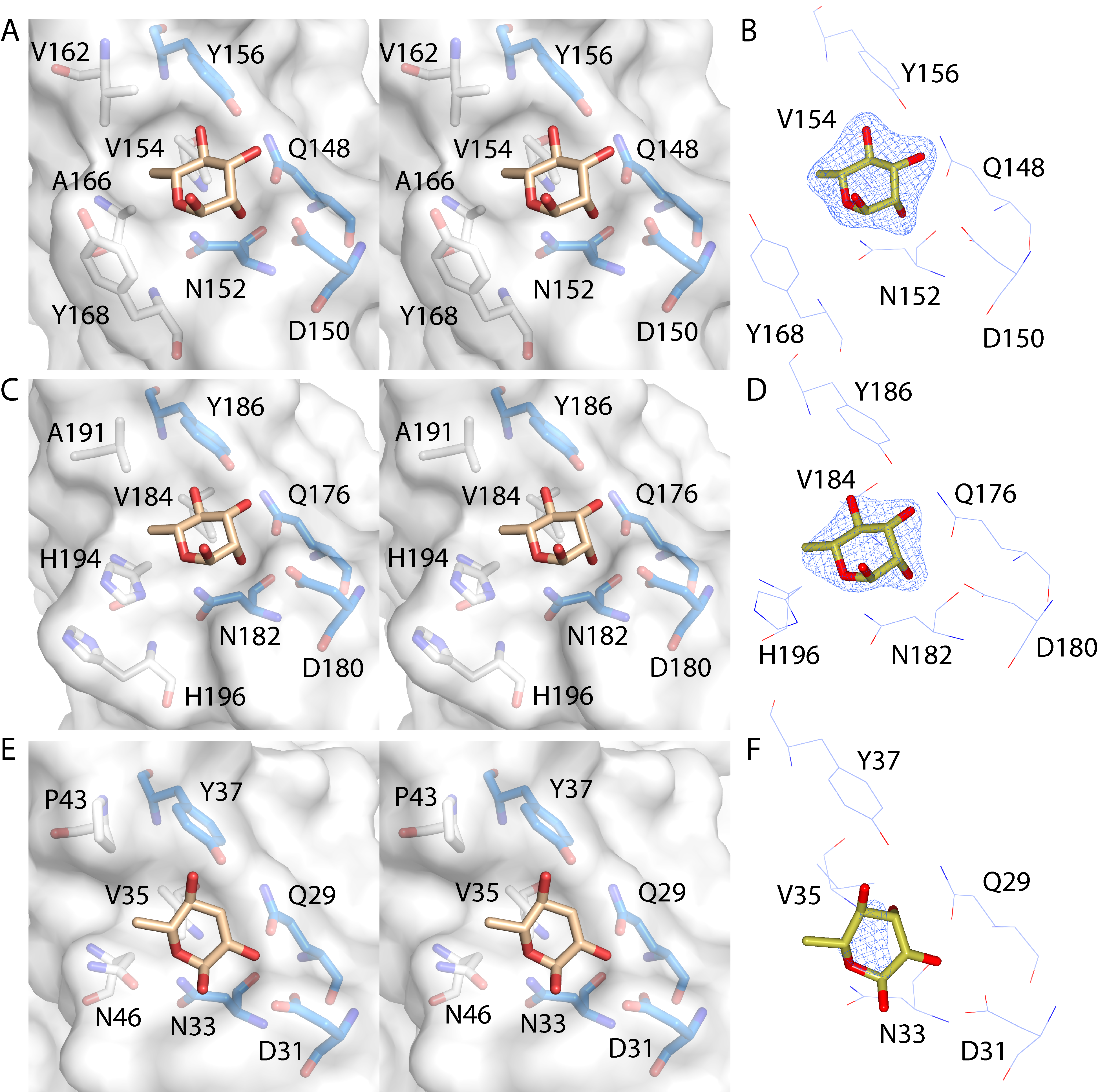

Supplement: Figure S5 — Coordination of d-rhamnose in C1, C2 and N2 binding sites of pyocin L1. (A) Stereo view of d-rhamnose coordination by binding site C1 (A), C2 (C) and N1 (E), from d-rhamnose soak data. Core binding motif residues (blue) and additional residues contributing to the pocket (white) are shown. Omit map density for d-rhamnose in binding site C1 (B), C2 (D), N1 (F) calculated by refinement of data from d-rhamnose soaked crystal with model built from unsoaked crystal. Density for all sites contoured to 0.15e/Å3. (TIF) [file ppat.1003898.s005.tif]

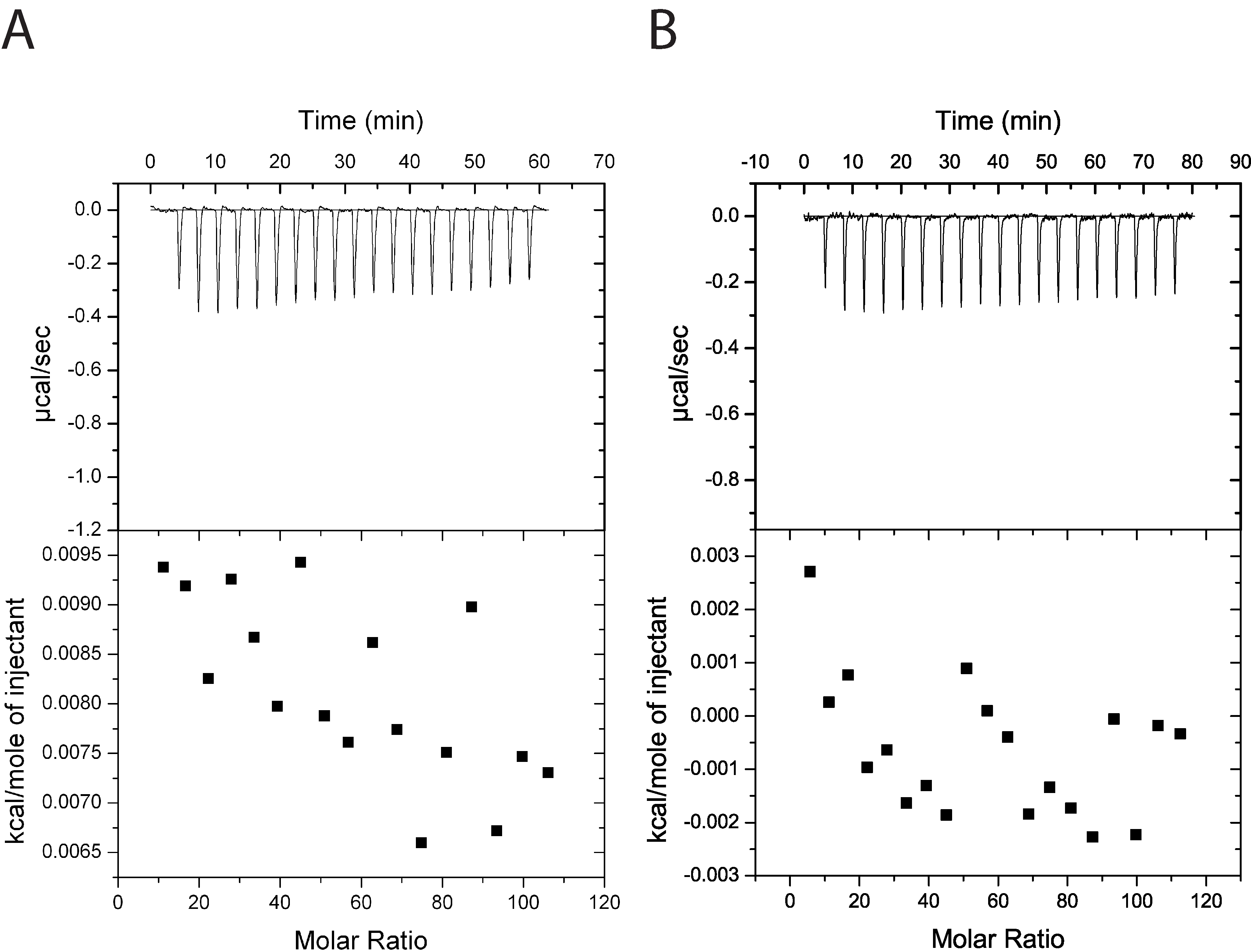

Supplement: Figure S7 — Putidacin L1 and pyocin L1 do not bind l-rhamnose. ITC isotherms of l-rhamnose (50 mM) titrated into putidacin L1 (A) and pyocin L1 (B) both at (0.1 mM). Binding is undetectable under these conditions. (TIF) [file ppat.1003898.s007.tif]
