# Supplementary material for: Lectin-Like Bacteriocins from Pseudomonas spp. Utilise D-Rhamnose Containing Lipopolysaccharide as a Cellular Receptor
Source: PLoS Pathog. 2014 Feb 6;10(2):e1003898. doi: 10.1371/journal.ppat.1003898 (PMC3916391; doi:10.1371/journal.ppat.1003898)
Supplement: Table S1 — Strains and plasmids used in this work. (PDF) [file ppat.1003898.s008.pdf]

Strain or plasmid	Genotype/Characteristics	Reference/Source	Pyocin L1 Sensitvity
P. aeruginosa			
PA01	Clinical isolate	[1]	6.25 µg/ml
wzm-G01::ISphoA/hah	PA01 wzm knockout	[2]	-
wzt-E12::ISphoA/hah	PA01 wzt knockout	[2]	-
E2	Environmental isolate	[3]	0.78 µg/ml
E2-M4	Pyocin L1 tolerant mutant	This study	2.6 mg/ml (very weak)
E2-M11	Pyocin L1 tolerant mutant	This study	2.6 mg/ml (very weak)
P1	Clinical isolate	This study	-
P2	Clinical isolate	This study	400 µg/ml
P3	Clinical isolate	This study	400 µg/ml
P4	Clinical isolate	This study	25 µg/ml
P5	Clinical isolate	This study	400 µg/ml
P7	Clinical isolate	This study	-
P8	Clinical isolate	This study	0.39 µg/ml
P9	Clinical isolate	This study	200 µg/ml
P10	Clinical isolate	This study	-
P11	Clinical isolate	This study	-
P12	Clinical isolate	This study	-
P13	Clinical isolate	This study	-
P14	Clinical isolate	This study	-
P15	Clinical isolate	This study	-
P16	Clinical isolate	This study	-
P17	Clinical isolate	This study	-
P18	Clinical isolate	This study	-
P19	Clinical isolate	This study	-
YH5	Clinical isolate	This study	50 µg/ml
PA14	Clinical isolate	[4]	-
PA7	Clinical isolate	[5]	-
PA62	Environmental isolate	[6]	-
MSH10	Environmental isolate	[3]	-
MSH3	Environmental isolate	[3]	-
C763	Clinical isolate	[6]	-
C1334	Clinical mucoid isolate	[6]	-
C1426	Clinical isolate	[6]	-
C1433	Clinical mucoid isolate	[6]	-
J1385	Clinical isolate	[7]	-
J1532	Clinical mucoid isolate	[7]	-
P. syringae			
pv. tomato DC3000	isolated from *Solanum lycopersicum*	BCCM	
pv. tomato NCPPB 1107	isolated from *Solanum lycopersicum*	NCPPB	
pv. tomato NCPPB 2563	isolated from *Solanum lycopersicum*	NCPPB	
pv. tomato NCPPB 3160	isolated from *Solanum lycopersicum*	NCPPB	
pv. coronafaciens LMG 5060	isolated from *Avena sativa*	BCCM	

pv. lachrymans LMG 5456	isolated from *Cucumis sativus*	BCCM	2.6 mg/ml (very weak)
pv. maculicola LMG 2208	isolated from *Brassica oleracea*	BCCM	
morsprunorum LMG2222	isolated from *Prunus avium*	BCCM	2.6 mg/ml (very weak)
pv. syringae LMG1247	isolated from *Syringa vulgaris,* type strain	BCCM	2.6 mg/ml (very weak)
pv. syringae LMG 5082	isolated from *Zea mays*	BCCM	2.6 mg/ml (very weak)
pv. syringae LMG 5084	isolated from *Pyrus communis*	BCCM	2.6 mg/ml (very weak)
P. carotovorum subsp. carotovorum			
LMG 2410	Isolated from *Cucumis sativus*	BCCM	-
LMG 2412	Isolated from *Hyacinthus orientalis*	BCCM	-
LMG 2442	Isolated from *Brassica oleracea*	BCCM	-
LMG 2444	Isolated from *Solanum tuberosum* (tuber soft rot)	BCCM	-
LMG 2913	Isolated from soil	BCCM	-
P. atrosepticum			
LMG 2374	Isolated from Apium graveolens var. dulce	BCCM	-
LMG 2375	Isolated from *Solanum tuberosum* (tuber soft rot)	BCCM	-
LMG 2386	Isolated from *Solanum tuberosum* (stem rot)	BCCM	-
LMG 2391	Isolated from soil	BCCM	-
SCRI 1043	Isolated from *Solanum tuberosum* (tuber soft rot)	SCRI	-
E. coli			
DH5α	F-, φ80dlacZΔM15, Δ(lacZYA-argF)U169, deoR, recA1, *endA1, hsdR17*(rk^-*,* mk^+*), phoA, supE44,* λ^-*, thi-1, gyrA96, relA1*	Invitrogen	-
BL21(DE3)pLysS	F^- *ompT hsdS* B (rB^-mB^-) *gal dcm* (DE3) pLysS (Cam^R)	Invitrogen	-
**Plasmids**			
pET21a	$Amp^{r,}$ cloning/expression vector, T7 promoter	Novagen	
pETPyoL1	771bp xhoI/NdeI fragment in pET21a	This study	
pETPL1	828bp xhoI/NdeI fragment in pET21a	This study	
