# Supplementary material for: Lectin-Like Bacteriocins from Pseudomonas spp. Utilise D-Rhamnose Containing Lipopolysaccharide as a Cellular Receptor
Source: PLoS Pathog. 2014 Feb 6;10(2):e1003898. doi: 10.1371/journal.ppat.1003898 (PMC3916391; doi:10.1371/journal.ppat.1003898)
Supplement: Text S1 — References for supplementary information. (DOCX) [file ppat.1003898.s009.docx]

Supplementary information.

References for table S1

1. Stover CK, Pham XQ, Erwin AL, Mizoguchi SD, Warrener P, *et al.* (2000) Complete genome sequence of *pseudomonas aeruginosa* PAO1, an opportunistic pathogen. Nature 406: 959-964.

2. Jacobs MA, Alwood A, Thaipisuttikul I, Spencer D, Haugen E, *et al.* (2003) Comprehensive transposon mutant library of *pseudomonas aeruginosa.* Proc Natl Acad Sci U S A 100: 14339-14344.

3. Wolfgang MC, Kulasekara BR, Liang XY, Boyd D, Wu K*, et al.* (2003) Conservation of genome content and virulence determinants among clinical and environmental isolates of *pseudomonas aeruginosa*. Proc Natl Acad Sci U S A 100: 8484-8489.

4. Rahme LG, Stevens EJ, Wolfort SF, Shao J, Tompkins RG, *et al*. (1995) Common virulence factors for bacterial pathogenicity in plants and animals. Science 268: 1899-1902.

5. Roy PH, Tetu SG, Larouche A, Elbourne L, Tremblay S, *et al.* (2010) Complete genome sequence of the multiresistant taxonomic outlier *pseudomonas aeruginosa* PA7. PLoS One 5: e8842.

6. Stewart L, Ford A, Sangal V, Jeukens J, Tucker N, *et al*. (2013) Draft genomes of twelve host adapted and environmental isolates of Pseudomonas aeruginosa and their position in the core genome phylogeny. (In preparation)

7. Terry JM, Pina SE, Mattingly SJ (1992) Role of energy-metabolism in conversion of nonmucoid *pseudomonas aeruginosa* to the mucoid phenotype. Infect Immun 60: 1329-1335.
